# Quantitative assessment of angiogenesis and pericyte coverage in human cell-derived vascular sprouts

**DOI:** 10.1186/s41232-016-0033-2

**Published:** 2017-01-18

**Authors:** Jan Eglinger, Haiko Karsjens, Eckhard Lammert

**Affiliations:** 1 Institute of Metabolic Physiology, Heinrich-Heine University, Düsseldorf, Germany; 2 Institute for Beta Cell Biology, Leibniz Center for Diabetes Research, German Diabetes Center (DDZ), Düsseldorf, Germany; 3 Current address: Friedrich Miescher Institute for Biomedical Research, Basel, Switzerland

**Keywords:** Angiogenesis, Pericytes, Sprouting, Image analysis, Morphometry

## Abstract

**Background:**

Pericytes, surrounding the endothelium, fulfill diverse functions that are crucial for vascular homeostasis. The loss of pericytes is associated with pathologies, such as diabetic retinopathy and Alzheimer’s disease. Thus, there exists a need for an experimental system that combines pharmacologic manipulation and quantification of pericyte coverage during sprouting angiogenesis. Here, we describe an in vitro angiogenesis assay that develops lumenized vascular sprouts composed of endothelial cells enveloped by pericytes, with the additional ability to comparatively screen the effect of multiple small molecules simultaneously. For automated analysis, we also present an ImageJ plugin tool we developed to quantify sprout morphology and pericyte coverage.

**Methods:**

Human umbilical vein endothelial cells and human brain vascular pericytes were coated on microcarrier beads and embedded in fibrin gels in a 96-well plate to form lumenized vascular sprouts. After treatment with pharmacologic compounds, sprouts were fixed, stained, and imaged via optical z-sections over the area of each well. The maximum intensity projections of these images were stitched together to form montages of the wells, and those montages were processed and analyzed.

**Results:**

Vascular sprouts formed within 4–12 days and contained a patent lumen surrounded by a layer of human endothelial cells and pericytes. Using our workflow and image analysis, pericyte coverage after treatment with various compounds was successfully quantified.

**Conclusions:**

Here we present a robust in vitro assay using primary human vascular cells that allows researchers to analyze the effects of multiple compounds on sprouting angiogenesis and pericyte coverage. Our ImageJ plugin offers automated evaluation across multiple different vascular parameters, such as sprout length, cell density, branch points, and pericyte coverage.

**Electronic supplementary material:**

The online version of this article (doi:10.1186/s41232-016-0033-2) contains supplementary material, which is available to authorized users.

## Background

Sprouting angiogenesis, the formation of new capillary branches from pre-existing blood vessels or lymphatic vessels, is required to build functional vascular networks, and altered sprouting angiogenesis is involved in many diseases [1].

Sprouting is induced by activation of endothelial cells (ECs) via growth factors such as vascular endothelial growth factor (VEGF), platelet-derived growth factor (PDGF), placental growth factor (PlGF), and hypoxia-inducible factor (HIF)-1α [2]. Activated ECs proliferate, extend into the surrounding tissue [3], and recruit pericytes to attach to the outer wall of the newly formed vessels [4]. The presence of pericytes on mature capillaries is required for stabilization of the endothelium and for regulation of blood flow, as observed in the blood–brain barrier [5, 6]. Pericytes are in close contact with ECs, as they extend long cytoplasmic processes across several ECs to encircle the EC-derived vascular tubes. Moreover, they share a basement membrane and physically interact via numerous contacts such as for example peg-socket junctions, adhesion plaques, or gap junctions [7]. Dysfunctional interplay between pericytes and the endothelium is frequently the cause or consequence of many diseases [8–10], resulting in increased vascular permeability and defective vessel maturation, which promote vessel leakage and inflammation [11]. Cancer cells, for instance, can induce detachment of pericytes from quiescent vasculature, thereby activating ECs to sprout into the surrounding tissue [12]. Pericyte detachment is also one of the first pathological hallmarks during diabetic retinopathy. The disease begins with a thickening of the vascular basement membrane, followed by a loss of pericytes and an increase in vascular permeability [13]. Ultimately, neovascularization causes hemorrhaging and vision loss [14]. On the contrary, after stroke, pericytes constrict around capillaries to decrease blood flow. When these pericytes die, the blood–brain barrier is disrupted, leading to progressive neuronal damage [15].

It is hypothesized that pericytes also play a regulatory role during angiogenesis [11, 16]. Our aim was to develop a method to assess pericyte coverage during sprouting angiogenesis in a defined human cellular system. We modified a sprouting assay that involves ECs and was previously used in a number of recent publications [17–19]. Nakatsu et al. described an optimized angiogenesis assay that leads to vascular sprouts with a defined lumen. This assay uses fibrin as an extracellular matrix and is characterized by the formation of vascular sprouts that harbor patent multi-cellular lumens and a basement membrane [20]. The sprouts undergo many critical steps that occur in vivo during angiogenesis. In order to mimic the in vivo vasculature as accurately as possible, we have modified their protocol to include human vascular pericytes [7]. After co-culturing ECs and pericytes, we verified that sprouts have lumens and that pericytes attach to the outer wall of vascular sprouts. Further, we adapted the original method for use in multi-well plates in order to simultaneously test and compare multiple treatments under reproducible conditions. Finally, for consistent analysis of sprout morphology and pericyte coverage, we developed an ImageJ plugin that measures sprout number, length and width, as well as cell density and pericyte coverage of vascular sprouts. While similar tools were published for standardized quantification of other angiogenesis assays, such as “Angiotool” for the quantification of retinal vascular networks [21], or “Aqual” for the aortic ring assay [22], no such standardized tool was previously available for the bead sprouting assay. Using our optimized analysis workflow, we tested more than 40 compounds for their biological effects on sprout morphology and pericyte coverage.

Here, we describe a standardized procedure of the bead sprouting assay using human umbilical vein ECs (HUVECs) and human brain vascular pericytes (HBVPs). To ensure comparability between experimental repetitions as well as between different researchers performing the experiments, we developed, using the plugin tool described below, a standardized automated quantitative analysis of developing angiogenic sprouts covered with pericytes.

## Methods

### Cell culture

We used HUVEC (Lonza) up to passage 6 [23], HBVP (ScienCell) were used up to passage 4, and Detroit-551 human skin fibroblasts (HSF; ATCC) up to passage 20. For experimental reproducibility, primary cells were used up to the passage number recommended by the respective supplier (Lonza, ScienCell, ATCC), as primary cells lose their identity and responsiveness to angiogenic stimuli at higher passages [23]. Cells were cultured in endothelial growth medium (EGM-2; Lonza) at least 24 h prior to starting the sprouting assay.

### Sprouting assay

A detailed step-by-step protocol for the sprouting assay is available in Additional files 1, 2 and 3. The procedure is based on a sprouting assay reported previously [17–19] that we extended to include pericytes and be adaptable for culture in a 96-well plate. In short, dextran-coated Cytodex-3 microcarrier beads (GE Healthcare) were incubated with HUVEC and HBVP in EGM-2 (Lonza) overnight. We found an endothelial-to-pericyte ratio of 10:1 to yield optimal pericyte coverage of developing sprouts in accordance with cell ratios found in tissues varying between 1:1 and 10:1 [7, 24]. Cell-coated beads were then embedded into fibrin gels by adding 90 μl beads in PBS (200 beads/ml) with 2.5 mg/ml fibrinogen (Sigma-Aldrich) to 8-μl thrombin (0.625 U/ml, Sigma-Aldrich) per well in a 96- well plate (μclear, Greiner Bio-One). The gels were overlaid with human skin fibroblasts at 1000 cells per well. As previously published, the cross-talk between ECs and fibroblasts, but not their direct contact, was shown to be required for the formation of stable lumenized sprouts [17]. Two hundred microliters of EGM-2 were subsequently added to each well. Plates were incubated at 37 °C, 5% CO_2_, and medium was changed every other day. After 6 days, when lumenized sprouts were observed, treatment was started by adding compounds at the respective concentrations to each well. Medium and compounds were changed every other day. The frequency of changing medium and compounds was chosen based on preliminary experiments that showed a significant decrease in sprout number and length upon treatment with the VEGFR inhibitor SU5416.


Additional file 1: Movie introducing the bead sprouting assay and our software analysis. (MOV 22013 kb)
Additional file 2: Movie demonstrating the analysis of human EC/pericyte-derived vascular sprouts. (MOV 4946 kb)


### Phalloidin staining

For assays containing ECs only, fibrin gels were fixed overnight using 4% paraformaldehyde in PBS. Subsequently, gels were washed with PBS, blocked with PBS containing 0.2% Triton X-100 and 1% BSA, and stained with Alexa Fluor-488-conjugated phalloidin and DAPI (for timing and details, see protocol in Additional file 3). After three washes (10 min in PBS), the plate containing the gels was imaged as described below.

### Immunostaining

Fibrin gels containing EC/pericyte sprouts were fixed using 4% paraformaldehyde in PBS. Subsequently, gels were washed with PBS, blocked with PBS containing 3% Triton X-100 and 1% BSA, incubated with primary antibodies (rabbit polyclonal anti-human Erg-1/2/3, Santa Cruz; mouse monoclonal anti-human NG2, Abcam) for at least 60 h, washed with PBS, and stained with secondary antibodies (Alexa Fluor 488 donkey anti-mouse IgG, Life Technologies; Cy3 AffiniPure donkey anti-rabbit IgG, Jackson ImmunoResearch), as well as Alexa Fluor 647-conjugated phalloidin and DAPI for at least 40 h (for timing and details, see protocol in Additional file 3). After three washes (PBS), the plate containing the gels was imaged as described below.

### Microscopy

We used an inverted microscope (Zeiss Axio Observer.Z1) with an automated stage to acquire multi-tile z-stacks (10-μm intervals) of stained sprouts, using a Zeiss 10×/NA0.45 objective lens. Both a Zeiss ApoTome system equipped with Colibri LED light source, as well as a Zeiss LSM 710 confocal microscope system were used for imaging. Images were acquired in grid positions and subsequently stitched using the Grid/Collection stitching plugin in Fiji [25], resulting in a continuous image coverage of 70% of each well (3 × 3 mm).

### Image analysis

Images were analyzed using the Fiji distribution of ImageJ [26] at version ImageJ 2.0.0-rc-15/1.49k; Java 1.6.0_65 (available as a Life-Line Version from https://imagej.net/Downloads). We developed a plugin for sprout analysis that is available from the *Angiogenesis* update site within ImageJ and is maintained to work with the newest version of Fiji (see https://imagej.net/Following_an_update_site for instructions how to install the plugin in Fiji). Documentation for our plugin is available at https://imagej.net/Sprout_Morphology. For each z-stack acquired, the maximum intensity projection (MIP) was created (see Additional file 4) and processed using our plugin. For each 96-well plate, representative MIPs were used to adjust analysis parameters to improve image clarity across the entire dataset. Using the provided script (see Additional file 5), the plugin was then run on every continuous image to consistently analyze the entire dataset.

### Statistical analysis

The results of the sprout analysis were normalized to either a PBS control or a DMSO control, depending on the solvent used to reconstitute each treatment compound (see Additional file 6: Table S1). Within one assay, the values for repeated conditions in separate wells were weighted according to the number of beads in each well. Significant increase or decrease of each measurement parameter at each treatment condition was determined using a one-sample *t* test.

## Results

### Lumen formation and pericyte coverage in multi-cellular sprouts

**Fig. 1:**
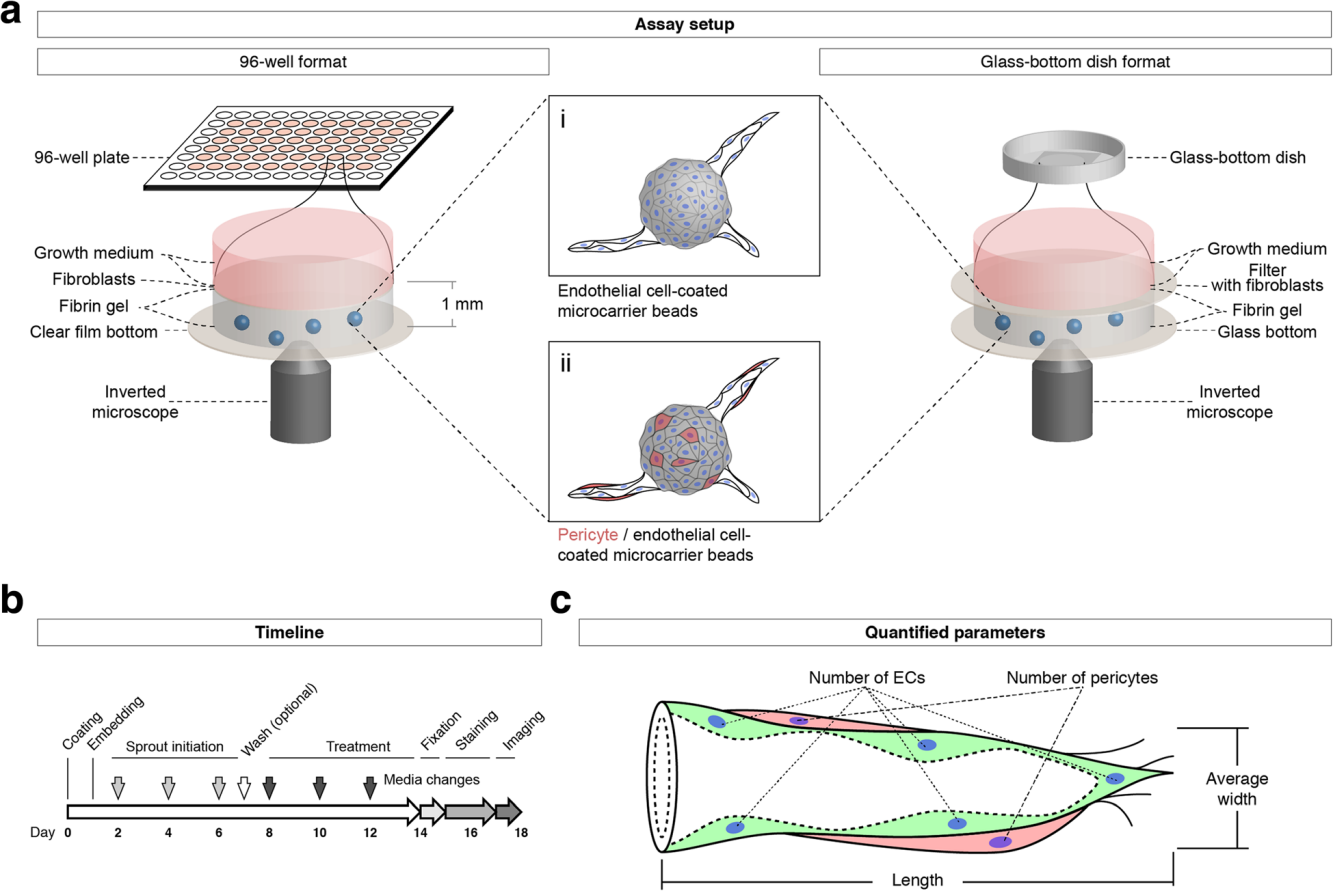
Setup and workflow of the EC/pericyte sprouting assay. **a** Schematic drawing of the assay setup: A 1:10 co-culture of pericytes (*red*) and endothelial cells are coated on microcarrier beads (centre) that are then embedded into fibrin gels in each of the inner 60 wells of a 96-well plate (*left*). Alternatively, cell-coated beads are embedded into fibrin gels in a glass-bottom culture dish (*right*). The fibrin gels are subsequently covered with a layer of fibroblast cells (*left*), which can also be placed onto filters (*right*) to facilitate removal and subsequent staining. Developing sprouts are cultured in growth medium containing the desired treatment, and they can be imaged using an inverted microscope. **b** Timeline of the assay procedure: after coating ECs or ECs/pericytes on microcarrier beads and embedding them in fibrin gels, sprouts are grown for up to 6 days, optionally supplied with 10-μM vanadate to accelerate sprout growth; after removal of vanadate by washing with growth medium, treatment with test compounds or growth factors can be pursued for another 6 days; for image analysis, sprouts are fixed, stained, and imaged using an inverted microscope. **c** Schematic representation of a sprout containing ECs (*green*) and pericytes (*red*), indicating the quantified sprout parameters

To assess vascular lumen formation within endothelial cell (EC)-derived vascular sprouts with and without pericytes, we seeded HUVEC in the presence or absence of HBVP onto microcarrier beads that subsequently were embedded into fibrin gels. In Fig. 1, a summary of the experimental design is shown, which can be performed in either 96-well plates to screen compounds (Fig. 1a, left side) or in glass-bottom dishes for staining with various different antibodies (Fig. 1a, right side). A timeline of the assay and the structure of a typical vascular sprout derived from human ECs and pericytes is also shown (Fig. 1b, c).

**Fig. 2:**
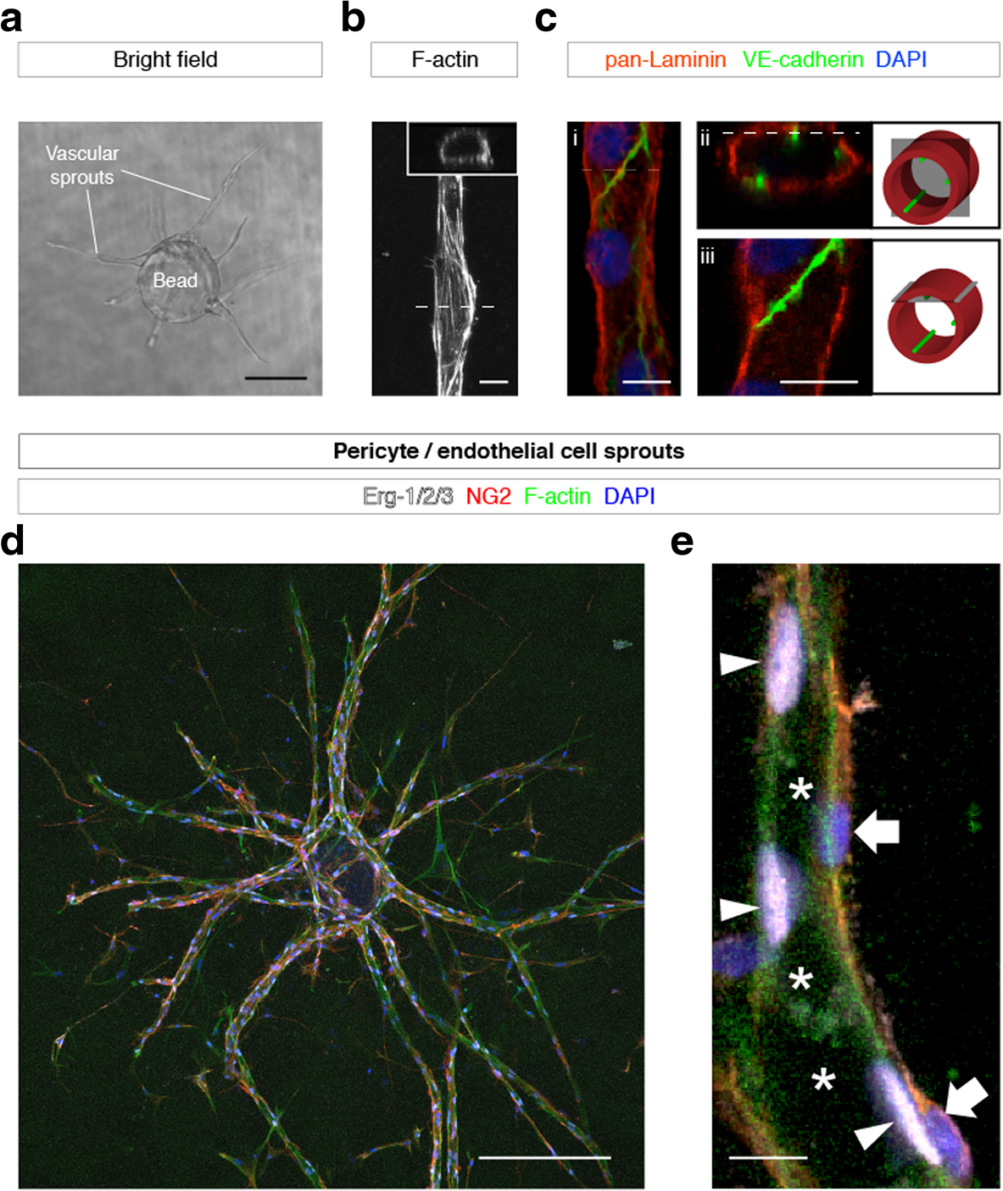
Validation of sprout morphology. **a** Bright field image of vascular sprouts growing out from an EC-coated bead. **b** Confocal micrograph of a vascular sprout stained for F-actin; a patent lumen is seen on an optical cross-section (*inset*). **c** Confocal micrographs of a vascular sprout stained for laminin (*red*), VE-cadherin (*green*), and nuclei (DAPI, *blue*); maximum intensity projection (*i*), optical cross-section (*ii*), and optical longitudinal section (*iii*); 3D configuration of the optical sections is depicted in simplified illustrations (*right*). **d**, **e** Confocal micrographs of human pericyte/EC-derived vascular sprouts stained for endothelial nuclei (Erg-1/2/3, *white*), pericytes (NG2, *red*), F-actin (phalloidin, *green*), and nuclei (DAPI, *blue*); maximum intensity projection showing a bead with connected sprouts (**d**), optical longitudinal section of a single sprout (**e**), showing the vascular lumen (*asterisks*), EC nuclei (*arrowheads*), and pericyte nuclei (*block arrows*). Scale bars, **a** 200 μm; **b**, **c** 10 μm; **d** 200 μm; **e** 10 μm

After an incubation of either 2 (with pericytes) or 4 days (without pericytes), the first sprouts formed a patent vascular lumen detectable by bright field transmission light microscopy (Fig. 2a). After fixation and staining of the F-actin cytoskeleton by fluorescently labeled phalloidin, a lumen was also detected in optical cross-sections of confocal z-stacks (Fig. 2b). Vascular sprouts were composed of multiple cells connected through VE-cadherin positive junctions and surrounded by a vascular basement membrane containing laminin (Fig. 2c). Pericytes aligned to the abluminal side of the vascular sprouts (Fig. 2d, e).

### Quantitative measurement of sprout morphology and pericyte coverage

**Fig. 3:**
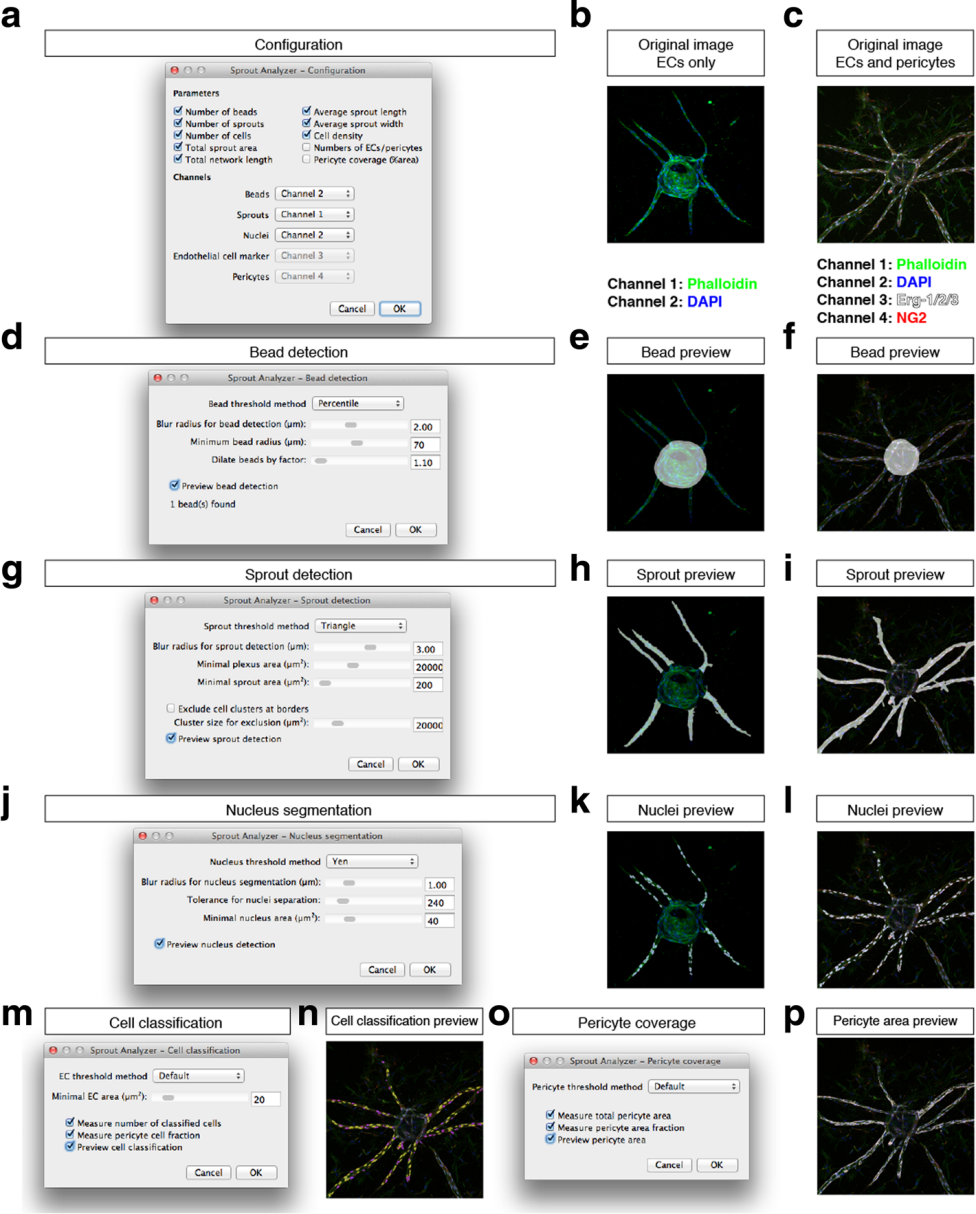
Configuration of the *Sprout Analyzer* plugin. **a** Dialog to configure output measurement parameters and to define input configuration, i.e., which image channel contains which staining information. **b** Example image showing a microcarrier bead with vascular sprouts stained for F-actin using phalloidin-AF488 (*green*) and nuclei using DAPI (*blue*). **c** Example image with EC/pericyte-derived sprouts stained for F-actin using phalloidin-AF488 (*green*), nuclei using DAPI (*blue*), EC nuclei using an anti-Erg-1/2/3 antibody (*white*), and pericytes using an anti-NG2 antibody (*red*). **d** Dialog to configure bead detection; when the preview checkbox is activated, a bead mask image (*white*) is overlaid onto the image (**e** and **f** for the original images in **b** and **c**, respectively). **g** Dialog to configure sprout detection; when the preview checkbox is activated, a sprout mask image (*white*) is overlaid onto the image (**h**, **i**). **j** Dialog to configure nucleus detection; when the preview checkbox is activated, a nucleus mask image (*white*) is overlaid onto the image (**k**, **l**). **m** Dialog to configure cell classification; when the preview checkbox is activated, a preview showing ECs (*yellow*) and pericytes (*magenta*) is overlaid onto the image (**n**). **o** Dialog to configure pericyte coverage measurement; when the preview checkbox is activated, a pericyte area mask image (*white*) is overlaid onto the image (**p**). For detailed instructions, see protocol in Additional file 3, Additional file 1: Video S1, and Additional file 2: Video S2

We developed a plugin for ImageJ to measure parameters of sprout morphology (Fig. 3). For image analysis with our plugin, MIPs of sprout images were created. The adjustment of analysis parameters was performed on a selected subset of images representative of the variability within the whole dataset. We adjusted the measurement parameters for each of the analysis steps (Fig. 3a–c), including the number of beads (Fig. 3d–f), number of vascular sprouts (Fig. 3g–i), total number of nuclei (Fig. 3j–l), and area covered with pericytes (Fig. 3m–p). The MIPs of whole well images from 96-well plates or single beads in glass-bottom dishes were analyzed using the ImageJ plugin we developed.

**Fig. 4:**
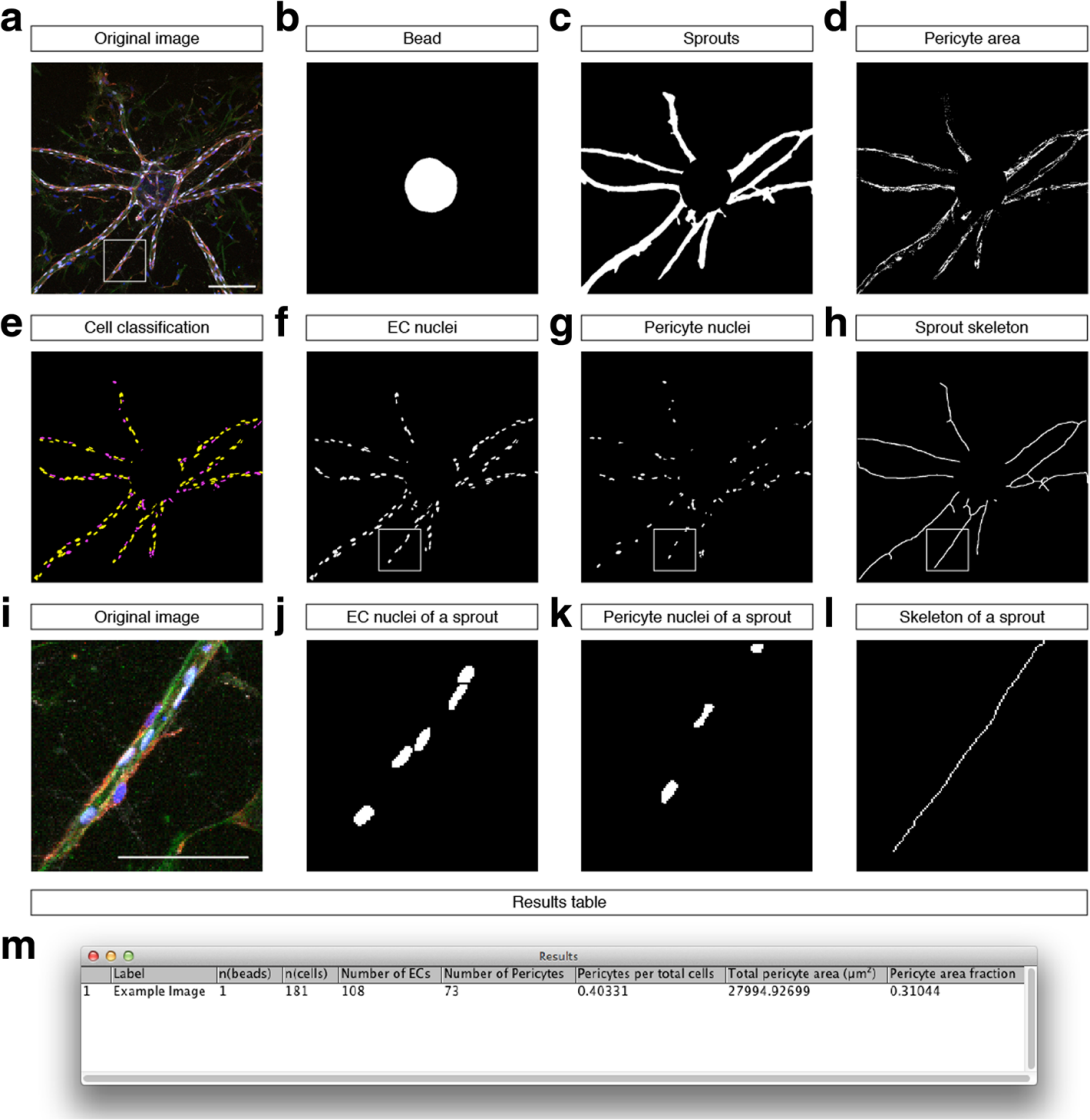
Automated multi-parametric image analysis of sprouting angiogenesis with pericytes. **a** Confocal micrograph (maximum intensity projection) showing a bead with sprouts stained for F-actin (*green*), nuclei (*blue*), endothelial nuclei (*white*), and pericytes (*red*). **b**–**d** Result images showing segmentation results (*white*): segmented bead (**b**), total sprout area (**c**) and pericyte area (**d**). **e** Cell classification result showing endothelial nuclei (*yellow*) and pericyte nuclei (*magenta*). **f**–**g** Result images from the cell classification showing EC nuclei (**f**) and pericyte nuclei (**g**). **h** Sprout skeleton for length measurements. **i**–**l** Magnified details of the marked region in (**a**, **f**–**h**). **m** Result table shown after running our plugin on the example image shown in **a**. Scale bars, **a**–**h** 200 μm; **i**–**l** 100 μm

An example of the produced image-based quantitation is shown in Fig. 4. Starting with the original input image (Fig. 4a), our plugin detects the bead (Fig. 4b) and the sprouts connected to it (Fig. 4c). The intensity of immunostaining against NG2, a pericyte marker, is used to segment pericyte area in the image (Fig. 4d). The presence or absence of Erg-1/2/3 staining is used to classify DAPI-positive cell nuclei into EC and pericyte-derived nuclei (Fig. 4e–g). For length measurements, the morphological skeleton of the sprouts is generated (Fig. 4h). The segmentation results are then used to measure morphological parameters of vascular sprouts, such as sprout number, length and width, as well as cell density, the ratio between ECs and pericytes, and the area of pericyte coverage (Fig. 4m).

### Assessment of small molecule modifiers of angiogenesis identifies specific modulators of pericyte coverage

Using our analysis workflow, we tested more than 40 compounds on their effect on sprout morphology and pericyte coverage (Additional file 6: Table S1). We selected in particular those compounds that target pathways reported to modulate angiogenesis (e.g., SU5416, a potent inhibitor of VEGFR-2 [27]; PDGFR inhibitors [10]; Y27632, a Rho kinase inhibitor [28]; a γ-secretase inhibitor (Notch signaling) [29]; and modulators of cAMP signaling [30]) in order to come up with a set of molecules to be used in a proof-of-concept study. The results are summarized in Additional file 7: Table S2 and Additional file 8: Table S3. In sprouts consisting of only ECs, we identified compounds that significantly decreased (11 compounds) or increased (6 compounds) sprout number (*p* < 0.05) with at least one of the concentrations used (Additional file 7: Table S2). Using a cut-off value of *p* < 0.01 (to reduce the chance of finding false positives), the number of compounds considered significantly decreasing or increasing sprout number was 6 and 3, respectively. Similarly, sprout length was significantly decreased with 12 (with *p* < 0.01, 5) compounds and increased with 3 compounds (with *p* < 0.01, 1). In the presence of pericytes, none of the compounds decreased the number of sprouts significantly with the concentrations used, and only 3 compounds significantly increased sprout number (with *p* < 0.01, 0; Additional file 8: Table S3). Sprout length was significantly decreased with 8 (with *p* < 0.01, 3) compounds and significantly increased with 2 (with *p* < 0.01, 0) compounds.

For EC-only sprouts, we measured sprout width and cell density as additional parameters. We found that 8 out of 41 compounds specifically and significantly (*p* < 0.05) change sprout width and/or cell density without affecting sprout number or length (Additional file 7: Table S2). Similarly, we found that 12 out of 42 compounds specifically changed pericyte coverage in human EC/pericyte-derived vascular sprouts (Additional file 8: Table S3).

## Discussion

The bead sprouting angiogenesis assay in its original form as described by Nakatsu et al. has been used in a number of publications to test the specific effects of agents (e.g., siRNAs, antibodies, chemical inhibitors) on angiogenesis, and to complement results from in vivo experiments [31–34]. However, until now, this assay has not included the important influence of pericytes on vascular sprout formation. We believe that the presented assay compares favorably with prior uses of published assays, since the co-culture of pericytes and ECs better mimics the in vivo vascular system. In addition, the three-dimensional bead angiogenic sprouting assay has been used with other cell types than HUVEC such as human lung ECs [35] and human lymphatic ECs [36].

The bead sprouting assay experimental design has several advantages over many comparable in vitro methods, namely, a defined cellular composition including ECs and pericytes. Based on a reproducible 3D environment, the assay produces multi-cellular lumenized sprouts that possess an abluminal basement membrane [20].

The assay reproduces the cellular mechanisms necessary to form lumenized tubes, in contrast to assays that focus solely on EC migration [37]. The new ImageJ plugin allows for straightforward quantification of pericyte coverage in addition to sprout morphology (see Additional file 2: Video S2). Future applications of this assay will benefit from the detailed quantitative analysis provided by our toolkit. Admittedly, a limitation of the assay is the absence of blood flow. However, methods providing a blood flow require a more complex setup, thereby limiting the sample size. A detailed comparison of assays highlighting their respective features is shown in Additional file 9: Table S4.

With the addition of pericytes, this sprouting angiogenesis assay is useful to measure effects of different compounds on pericyte coverage. In our assay, we often observed vascular sprouts with leading ECs and pericytes in close vicinity to the endothelium, covering the abluminal side of sprouts with long cellular protrusions (Fig. 4i–l). The observed sprout morphology is characteristic of pericyte-covered sprouting capillaries in vivo [6, 7, 10].

Within our test compounds, the number of compounds that decreased sprout number and length was higher than those that increased these two parameters. On an evolutionary-optimized cellular mechanism like angiogenesis, it is not surprising to find more inhibiting than activating factors. In addition, we observed that the presence of pericytes on the vessel walls stabilized vascular sprouts; in the EC/pericyte sprouts, fewer compounds were able to decrease sprout number, although they were used at the same concentrations in both experiments.

It should be noted that our sprouting assay, while recapitulating the in vivo morphology of pericyte-wrapped sprouts, suffers from the natural biological variability present in primary human cells. A careful analysis of its statistical power should therefore be performed when designing larger studies using this assay to draw meaningful conclusions. Our proof-of-concept study is limited by its small sample size, since no robotic platform for high-throughput screening was used. However, when comparing groups of compounds with shared properties, we observed similar behaviors. For example, we observed that compounds regulating intracellular cyclic adenosine monophosphate (cAMP) levels or activating protein kinase A (i.e., 6-Bnz, Forskolin, IBMX, Sp-cAMPS) tended to increase sprout width in our assay (see Additional file 7: Table S2). In addition, the assay setup also allows measuring the number of branch points per sprout, in particular when sprouts are given enough time to form branched networks and a larger sample size is used. Therefore, we also added an option to measure the number of branch points in our plugin (Additional file 10: Figure S1), based on the “Analyze Skeleton” plugin included in Fiji [38].

The ImageJ plugin we developed provides a comprehensive quantification of morphological parameters in vascular sprouts. By providing an analysis specific to the bead sprouting assay, it helps researchers using this assay to get useful measurements, while still offering flexibility in the choice of parameters as well as the possibility of adaptations, as its source code is fully available.

## Conclusions

Our methodology allows for quantitative assessment of morphology and pericyte coverage in vascular sprouts developed from human endothelial cells and human pericytes in vitro. We were also able to observe some of the expected effects of anti-angiogenic substances, such as those of VEGF and PDGF receptor inhibitors. The in vitro bead sprouting angiogenesis assay with pericytes therefore represents a novel, helpful addition to various in vivo experiments. Our plugin for ImageJ allows to quantify the results of this assay and is a novel and freely available quantification tool in the field of digital pathology.

## Additional files

Additional file 3: Step-by-step protocol for the pericyte sprouting assay. This protocol contains several options of performing the bead sprouting assay, e.g., with or without pericytes, in a glass-bottom dish or a 96-well plate format, and with different stainings. (DOCX 132 kb)

Additional file 4: ImageJ macro to create maximum intensity projections. ImageJ macro (ijm) format; open the file using ImageJ, e.g., by dragging onto the ImageJ main window. (IJM 1 kb)

Additional file 5: ImageJ macro to process image folders. ImageJ macro (ijm) format; open the file using ImageJ, e.g., by dragging onto the ImageJ main window. (IJM 1 kb)

Additional file 6: Table S1. List of compounds and concentrations used. The compounds were used at two concentrations, indicated in the columns 10 × IC_50_ and 100 × IC_50_, respectively. The values were chosen according to published values for the half maximal inhibitory concentrations of the inhibitors with respect to their target molecules in vitro or in accordance with literature using the respective compounds in a similar assay setup. Literature references for each compound are listed in the reference column. (DOCX 193 kb)

Additional file 7: Table S2. Results of a pilot screen for EC-derived vascular sprouts. Changes considered significant with a cut-off *p* value of *p* **<** 0.05 (two-sided one-sample *t* test compared to solvent control) are colored in red and green to indicate decrease and increase compared to controls, respectively. *N* = 3 assays with 8 wells per compound each. (PDF 66 kb)

Additional file 8: Table S3. Results of a pilot screen for EC/pericyte-derived vascular sprouts. Changes considered significant with a cut-off *p* value of *p* **<** 0.05 (two-sided one-sample *t* test compared to solvent control) are colored in red and green to indicate decrease and increase compared to controls, respectively. *N* = 3 assays with 8 wells per compound each. (PDF 67 kb)

Additional file 9: Table S4. Comparison of in vitro and ex vivo assays. Comparison of in vitro and ex vivo assays to study sprouting angiogenesis, EC migration, and pericyte coverage. The method presented here is highlighted in green. Symbols used are the following: +, possible/present; −, not possible/not present; n.s., not shown. (DOCX 148 kb)

Additional file 10: Figure S1. Quantification of branching level. (a) Confocal micrograph (maximum intensity projection) of a sprouting vascular plexus growing from four microcarrier beads, stained for F-actin (green) and nuclei (red). (b) Result image showing bead (dark grey) and sprout (light grey) segmentation. (c) Result image showing the sprout skeletons; branch points have been highlighted in red. (d) The option to measure branching level (red box) is available in the configuration dialog. (e) Result table reporting the average number of branch points per sprout (avg n(branch points), red box). (PNG 375 kb)

## Abbreviations

EC: Endothelial cell
EGM-2: Endothelial growth medium-2
HBVP: Human brain vascular pericytes
HIF-1α: Hypoxia-inducible factor 1α
HSF: Human skin fibroblasts
HUVEC: Human umbilical vein endothelial cells
MIP: Maximum intensity projection
PDGF: Platelet-derived growth factor
PlGF: Placental growth factor
VEGF: Vascular endothelial growth factor
