# Supplementary material for: Quantitative assessment of angiogenesis and pericyte coverage in human cell-derived vascular sprouts
Source: Inflamm Regen. 2017 Jan 18;37:2. doi: 10.1186/s41232-016-0033-2 (PMC5725907; doi:10.1186/s41232-016-0033-2)
Supplement: Supplementary file 3 — Step-by-step protocol for the pericyte sprouting assay. This protocol contains several options of performing the bead sprouting assay, e.g., with or without pericytes, in a glass-bottom dish or a 96-well plate format, and with different stainings. (DOCX 132 kb) [file 41232_2016_33_MOESM3_ESM.docx]

**MATERIALS**

**REAGENTS**

- Human Umbilical Vein Endothelial Cells (HUVECs), pooled and cryopreserved in EGM-2 (Lonza, cat. no. C2519A)
- Detroit-551 Human Skin Fibroblasts (HSF; ATCC, cat. no. CCL-110)
- Human Brain Vascular Pericytes (HBVP; ScienCell, cat. no. 1200)
- EGM-2 BulletKit (containing FGF2 and VEGF-A; Lonza, cat. no. CC-3162)
- EGM-2 MV BulletKit (Lonza, cat. no. CC-3202)
- Minimum Essential Medium (MEM; Life Technologies, cat. no. 11095-080)
- Pericyte Medium (PM; ScienCell, cat. no. 1201)
- Fetal bovine serum (FBS; Life Technologies, cat. no. 10270-106)
- Dulbecco’s Phosphate Buffered Saline (DPBS; Life Technologies, cat. no. 14190-094)
- Cytodex 3 microcarrier beads (GE Healthcare, cat. no. 17-0485-01)
- Fibrinogen type I from bovine plasma (Sigma-Aldrich, cat. no. F8630)
- Thrombin from bovine plasma (Sigma-Aldrich, cat. no. T3399)
- Aprotinin from bovine lung (Sigma-Aldrich, cat. no. A1153)
- Trypsin/EDTA, UV-inactivated (PAA Laboratories, cat. no. L11-660)
- VEGF-C, recombinant (R&D systems, cat. no. 2179-VC-025)
- FGF2 (bFGF), recombinant (Miltenyi Biotec , cat. no. 130-093-839)
- Sodium ortho-vanadate (Sigma-Aldrich, cat. no. S6508)
- SU5416 (Sigma-Aldrich, cat. no. S8442)
- Dimethyl sulfoxide (DMSO; Sigma-Aldrich, cat. no. D8418)
- Paraformaldehyde solution 4% in PBS (Santa Cruz Biotechnology, cat. no. sc-281692)
- Triton X-100 (Sigma-Aldrich, cat. no. T8787)
- Bovine serum albumin (BSA; AppliChem, cat. no. A1391)
- Alexa Fluor 488 phalloidin (Life Technologies, cat. no. A12379)
- Alexa Fluor 647 phalloidin (Life Technologies, cat. no. A22287)
- 4',6-Diamidino-2-Phenylindole, Dilactate (DAPI; Molecular Probes, cat. no. D3571)
- Mounting medium (e.g. Fluoroshield; Sigma-Aldrich, cat. no. F6182)
- Rabbit polyclonal anti-human Erg-1/2/3 antibody (C-20; Santa Cruz Biotechnology, cat. no. sc-353)
- Mouse monoclonal anti-human NG2 antibody [9.2.27] (abcam, cat. no. ab78284)
- Mouse anti-VE-cadherin monoclonal antibody (Merck Millipore, cat. no. MAB1989)
- Rabbit anti-Laminin antibody (Sigma, cat. no. L9393)
- Alexa Fluor 488 donkey anti-mouse IgG (Life Technologies, cat. no. A21202)
- Cy3 AffiniPure donkey anti-rabbit IgG (Jackson ImmunoResearch, cat. no. 711-165-152)
- Alexa Fluor 647 donkey anti-rabbit IgG (Life Technologies, cat. no. A31573)

**EQUIPMENT**

- Cell culture incubator (humidified, 37 °C, 5% CO_2_)
- Cell culture hood with laminar flow and UV light
- Vacuum pump and tubing
- Centrifuge with a swing-bucket rotor
- Glass Pasteur pipettes (e.g. Brand, cat. no. 747720)
- Flat-bottom 96-well plates with lid (µClear; Greiner Bio-One, cat. no. 655090)
  **CRITICAL** For optimal imaging, use plates with a thin bottom (<200 µm)
- Cell-culture flasks, 25 cm^2^ (e.g. Sarstedt, cat. no. 83.1810.002)
- Cell-culture flasks, 75 cm^2^ (e.g. Sarstedt, cat. no. 83.1811.002)
- 5 ml round bottom polystyrene test tubes (Falcon; Corning, cat. no. 352054)
- 14 ml round bottom polypropylene (PP) tubes (Greiner Bio-One, cat. no. 187261)
- 15 ml conical PP tubes (Sarstedt, cat. no. 62.554.502)
- Battery-driven tube rotator (e.g. MACSmix Tube Rotator; Miltenyi Biotec, cat. no. 130-090-753)
- Laboratory film (e.g. Parafilm M; VWR, cat. no. 291-1214)
- Multi-step pipette (e.g. Eppendorf Multipette M4, cat. no. 4982000012)
- Combitips 0.2 ml (Eppendorf, cat. no. 0030089626)
- Combitips 1 ml (Eppendorf, cat. no. 0030089642)
- 300 µl multi-channel pipette (e.g. Pipetman Ultra Multichannel; Gilson, cat. no. F21042)
- Glass bottom culture dishes with No. 0 coverglass (MatTek, cat. no. P35G-0-14-C)
- Millicell cell culture inserts, 0.4 µm pore size (Merck Millipore, cat. no. PICM0RG50)
- Cell counting chambers (e.g. Fast Read; BioSigma, cat. no. BVS100) or a hemocytometer
- 0.45 µm sterile filters (VWR, cat. no. 514-0063)
- 10 ml plastic syringes (BD, cat. no. 300912)
- Cover glasses (e.g. Zeiss, cat. no. 474030-9010-000)
- Plastic tray, 12 cm × 16 cm × 4 cm
- Microscope (e.g. Zeiss AxioObserver.Z1 equipped with a 10x/0.45 objective lens and a motorized stage)
- Multi-well plate frame (e.g. Zeiss Universal Mounting Frame K-M, cat. no. 000000-1272-644)
- Fiji (to be downloaded from *http://fiji.sc/Downloads*)

**REAGENT SETUP**

**Endothelial growth medium (EGM-2)**
Supplement the growth media according to the supplier’s instructions before use.

**Fibroblast growth medium (MEM / 10% FBS)**
Supplement MEM with 10% FBS before use.

**Pericyte medium (PM)**
Supplement PM with 10% FBS before use.

**Endothelial cells**Human Umbilical Vein Endothelial Cells (HUVECs) should be grown to about 80% confluency in EGM-2 before starting this assay. A 75 cm^2^ flask yields about 1-2 × 10^6^ cells in our hands, which is sufficient for at least two parallel coatings. HUVECs between passages 2 and 8 should be used.

**Pericytes**Grow Human Brain Vascular Pericytes (HBVP) in PM to about 80% confluency. A 25 cm^2^ flask yields about 5 × 10^5^ cells in our hands. HBVP lower than passage 6 should be used.

**Fibroblasts**
Grow Human Skin Fibroblasts (HSF) in fibroblast growth medium to about 80% confluency.

**Microcarrier beads**Suspend 1 g Cytodex 3 microcarrier beads in 50 ml DPBS for soaking and equilibration, as recommended by the supplier. Wash twice with DPBS, then autoclave. The microcarrier solution can be kept at room temperature for one year and autoclaved again when necessary.

**Thrombin solution**
Dissolve thrombin at 50 U/ml in DPBS. Make aliquots of 0.5 ml each. Aliquots can be stored at -20 °C. Avoid repeated freezing and thawing.

**Aprotinin solution**
Dissolve aprotinin at 4 U/ml in ddH_2_O. Sterile filter the solution and make aliquots of 0.5 ml each. Aliquots can be stored at -20 °C.

**0.2% Triton blocking solution**
1% BSA, 0.2% Triton-X100 in DPBS (should be freshly prepared)

**3% Triton blocking solution**
1% BSA, 3% Triton-X100 in DPBS (should be freshly prepared)

**DAPI**
Reconstitute DAPI at 5 mg/ml in H_2_O.

**EQUIPMENT SETUP**

**Microscope**For imaging multi-well plates, use a suitable frame insert. Because of the wide variety of microscopes used in scientific laboratories, we can not give more specific advice how to set up the respective system.

**Software**Install Fiji (http://fiji.sc) by downloading and installing the files specific to your platform. Then, run the ImageJ updater via *Help > Update Fiji*, click on “*Manage update sites”* and add the update site “Angiogenesis” by activating its checkmark. This will install the “Sprout Analyzer” plugin we developed. Start the plugin by running the *Analyze > Sprout Morphology* command from the menu.

**PROCEDURE**

**Prepare fibroblasts TIMING 5 min (at the day of bead coating)**

1. Warm endothelial growth medium (EGM-2), DPBS and trypsin-EDTA at 37 °C in a water bath.
2. Remove and discard the growth media from a flask with 80% confluent HSF (see REAGENT SETUP; for the choice of feeder cells, see the “Experimental design” section), and replace with pre-warmed EGM-2. Incubate overnight at 37 °C, 5% CO_2_ in a cell culture incubator.

**Bead coating TIMING 4.5 h**

1. Transfer 80 µl of microcarrier bead suspension into a 5 ml round-bottom polystyrene tube and add 3 ml EGM-2. Let the beads sediment without shaking the tube.
2. The assay can be performed with pericytes and endothelial cells (ECs), or with ECs only. Use option A to test endothelial cell sprouting; follow option B to test effects on pericyte coverage of endothelial cell sprouts.
   1. **Coating of ECs onto microcarrier beads**
      1. Harvest 80% confluent HUVECs (see Reagent setup) by removing the media, rinsing with pre-warmed DPBS, adding 1 ml of trypsin-EDTA (for a 75 cm^2^ flask) and incubating at 37 °C for a few minutes to loosen the cells. Tap the side of the flask to fully detach the cells.
      2. Add 5 ml EGM-2, gently pipette the solution up and down, and transfer the cell suspension into a sterile 15 ml PP tube.
      3. Count the cells by transferring 10 µl of the cell suspension into a counting chamber or a hemocytometer.
      4. Centrifuge the cells at 200*g* for 5 min in a centrifuge with a swing-bucket rotor.
      5. Aspirate and discard the supernatant and resuspend the cells in EGM-2 at a concentration of 5 × 10^5^ cells per 1 ml.
      6. Carefully aspirate and discard the supernatant of the tube containing the sedimented beads (Step 3), leaving about 0.5 ml EGM-2 in the tube.
      7. Add 1 ml (5 × 10^5^ cells) of the cell suspension (Step 8) to the beads, close the lid halfway and shake well, but without inverting the tube.
   2. **Coating of pericytes and ECs onto microcarrier beads**
      1. Harvest both HUVECs and HBVPs at 80% confluency by removing the media, rinsing with pre-warmed DPBS, adding 1 ml of trypsin-EDTA (for a 75 cm^2^ flask) and incubating at 37 °C for a few minutes to loosen the cells. Tap the side of the flask to fully detach the cells.
      2. For both cell types, add 5 ml EGM-2, gently pipette the solution up and down, and transfer the cell suspensions into a sterile 15 ml PP tube each.
      3. Count the cells by transferring 10 µl of each cell suspension into a counting chamber or a hemocytometer.
      4. Centrifuge the cells at 200*g* for 5 min in a centrifuge with a swing-bucket rotor.
      5. Aspirate and discard the supernatant and resuspend the cells in EGM-2 at a concentration of 5 × 10^5^ cells per 1 ml.
      6. Carefully aspirate and discard the supernatant of the tube containing the sedimented beads (Step 3), leaving about 0.5 ml EGM-2 in the tube.
      7. Add 0.9 ml (4.5 × 10^5^ cells) of the HUVEC suspension and 0.1 ml (5 × 10^4^ cells) of the HBVP suspension to the beads, close the lid halfway and shake well, but without inverting the tube.
3. Coating of cells onto microcarrier beads requires agitation in regular time intervals. This can be done manually (option A) or with a suitable rolling device that can be placed into an incubator (option B).
   1. **Manual procedure TIMING 4 h**
      1. Place the bead-cell suspension into the incubator at 37 °C, 5% CO_2_. Shake the tube every 20 min for a time period of 4 h, taking care to raise all sedimented beads.

**CRITICAL STEP** To achieve a homogenous coating, it is important that the beads are mixed well and in regular intervals at least 12 times.

- - 1. After 4 h incubation with regular mixing, transfer the coated beads into a 25 cm^2^ flask with additional 7 ml EGM-2.

**CRITICAL STEP** Avoid damaging the coated beads by gentle aspiration.

- 1. **Automated shaking incubation TIMING 4 h**
     1. Fill the tube containing the cell/bead suspension with EGM-2 and close the lid tightly, leaving as little air as possible in the tube.
     2. Mount the tube containing the bead-cell suspension into a battery-driven roller device (e.g. MACSMix), switch to interval rotation mode (30 s rotation, 5 min 30 s pause) and position the roller in a 37 °C, 5% CO_2_ cell culture incubator.
     3. After 4 h rotation in the incubator, dismount the tube and transfer the coated beads into a 25 cm^2^ flask with additional 5 ml EGM-2

**CRITICAL STEP** Avoid damaging the coated beads by gentle aspiration.

1. Observe the coated beads under a low magnification microscope. They should have a golf ball-like appearance, with ECs or ECs/pericytes homogenously covering the bead surface.
2. Incubate overnight at 37 °C, 5% CO_2_.

**Fibrin gel embedding TIMING ~3 h**

1. Warm EGM-2, DPBS and trypsin-EDTA at 37 °C in a water bath.
2. Prepare a 2.5 mg/ml fibrinogen solution in DPBS. A total volume of 6 ml is required per 96-well plate, or 200 µl per glass-bottom dish. Incubate at 37 °C for 20 min to dissolve.
3. Thaw an aliquot of thrombin and aprotinin each (see REAGENT SETUP).
4. Transfer the EC-coated beads (Step 14) into a sterile 14 ml round-bottom PP tube. Rinse the flask with 4 ml EGM-2 and transfer to the same tube to recover as many beads as possible.
5. Let the beads sediment for about 2 min, then carefully aspirate and discard the supernatant, leaving about 0.5 ml in the tube to avoid aspirating beads.
6. Wash the beads by adding 5 ml of prewarmed EGM-2.
7. Let the beads sediment again for about 2 min, then aspirate and discard the supernatant, leaving as little growth medium as possible covering the beads.
8. Remove the plunger of a 10 ml syringe, connect the syringe to a 0.45 µm sterile filter, and pour the fibrinogen solution into the syringe. With the help of the plunger, filter the entire solution directly onto the microcarrier beads.
9. Add 37.5 µl aprotinin solution (see Reagent Setup) per ml of fibrinogen/microcarrier bead suspension, and mix carefully by pipetting up and down.
10. EC- or EC/pericyte-coated beads can be embedded using option A for analysis of many compounds and repeated measurements in a 96-well plate, or option B for investigation of few treatment conditions with subsequent immunostaining.
    1. **Embedding cell-coated beads into fibrin gels in a 96-well plate TIMING ~3 h**
       1. Prepare a 96-well plate by labelling the inner 60 wells that will be used for the assay.
       2. Using a multi-step pipette, transfer 8 µl of thrombin solution into each of the 60 inner wells of the 96-well plate.
       3. Transfer 90 µl of the bead-fibrinogen suspension (from Step 23) into each of the 60 inner wells of the plate.

**CRITICAL STEP** We recommend to use 1 ml Combitips (see Materials section) and pipet quickly one row of 10 wells at a time. This avoids sedimentation of the beads during prolonged periods of pipetting and thereby ensures an even distribution of beads into the wells. By pipetting into the center of the well without touching the plate, it is assured that fibrinogen and thrombin mix well and reproducibly.

- - 1. Let the gels solidify for about 5 min at room temperature, then keep them at 37 °C to avoid damage to the endothelial cells.
    2. Harvest feeder cells (from Step 2) by removing the media, rinsing with DPBS, adding 1 ml of trypsin-EDTA (for a 25 cm^2^ flask) and incubating at 37 °C for a few minutes to release the cells. Tap the side of the flask to fully detach the cells.
    3. Add 5 ml EGM-2, gently pipette the cell suspension up and down, and transfer it into a sterile 15 ml PP tube.
    4. Count the cells by transferring 10 µl of the cell suspension into a counting chamber or a hemocytometer.
    5. Centrifuge the cells at 200*g* for 5 min in a centrifuge with a swing-bucket rotor.
    6. Aspirate and discard the supernatant and resuspend the cells in EGM-2 at a concentration of 15,000 cells per 1 ml.
    7. Distribute the feeder cell suspension carefully onto the polymerized fibrin gels by pipetting 200 µl (3,000 cells) per well.
    8. Incubate overnight at 37 °C, 5% CO_2_.
  1. **Embedding and filter setup in a glass-bottom culture dish TIMING ~3 h**
     1. Pipet 20 µl of thrombin solution onto the glass of a glass-bottom culture dish.
     2. Pipet 180 µl of the bead-fibrinogen suspension onto the culture dish and mix by carefully moving the pipet tip through the solution.
     3. Immediately place a Millicell cell culture insert on top of the fibrin gel and let the gel solidify for about 5 min at room temperature.
     4. Harvest feeder cells (from Step 2) by removing the media, rinsing with DPBS, adding 1 ml of trypsin-EDTA (for a 75 cm^2^ flask) and incubating at 37 °C for a few minutes to loosen the cells. Tap the side of the flask to fully detach the cells.
     5. Add 5 ml EGM-2, gently pipette the cell suspension up and down, and transfer it into a sterile 15 ml PP tube.
     6. Count the cells by transferring 10 µl of the cell suspension into a counting chamber or a hemocytometer.
     7. Centrifuge the cells at 200*g* for 5 min in a centrifuge with a swing-bucket rotor.
     8. Aspirate and discard the supernatant and resuspend the cells in EGM-2 at a concentration of 15,000 cells per 1 ml.
     9. Seed feeder cells by pipetting 1 ml cell suspension (15,000 cells) onto the filter insert.
     10. Carefully add 2 ml EGM-2 to the side of the culture dish to also fill the space below the filter insert.
     11. Incubate overnight at 37 °C, 5% CO_2_

**Sprout initiation (optional) TIMING variable, ~4-8d**

1. Change growth medium every other day. Carefully aspirate the medium above the fibrin gel, leaving only a small amount of liquid covering the gel.
2. Refill each well with a suitable amount of pre-warmed EGM-2. To accelerate sprout growth as suggested for quantitative analysis, refill each well with a suitable amount of pre-warmed EGM-2 containing 10 µM sodium ortho-vanadate.
3. Incubate the fibrin gels for two days at 37 °C, 5% CO_2_. Repeat steps 25 and 26 every second day. Monitor the progress of sprouting using a bright-field microscope.
4. One day before start of the treatment, wash the samples with EGM-2 without vanadate.

**Treatment TIMING variable, ~2-6 d**

1. Apply the treatment solutions and controls to the fibrin gels. Follow option A for 96-well plate format, or option B for glass-bottom dishes.
   1. **Treatment in 96-well format**
      1. Prepare the required treatment solutions at 10 × the desired final concentration. For the negative control treatment for example, prepare a 40 µM solution of SU5416 in 0.2% DMSO in H_2_O to achieve a final treatment concentration of 4 µM.
      2. Carefully aspirate the medium above the fibrin gel in each well, leaving only a small amount of liquid covering the gel.

**CRITICAL STEP** Avoid destroying the gel or the fibroblast layer by aspirating carefully.

- - 1. Using a multi-channel pipet, carefully add 180 µl of new media to each well.
    2. Add 20 µl of 10 × concentrated treatment solutions to each well and carefully mix by pipetting up and down twice.
    3. Incubate the plate at 37 °C, 5% CO_2_ for two days. Repeat steps (i) to (iv) every other day for the desired period of treatment. A treatment length of 2 to 6 days is recommended. Monitor sprout growth regularly using a bright-field microscope.
  1. **Glass-bottom dish format**
     1. Aspirate the growth medium from above the filter insert and around it.
     2. Prepare 3 ml growth media containing the desired concentration of treatment agent. For the negative control treatment for example, prepare 3 ml of 4 µM SU5416 in EGM-2.
     3. Add 3 ml of growth medium containing the treatment agent to each culture dish. First add enough media on top of the filter to cover the surface, then carefully add the remaining media into the space between filter insert and culture dish. The liquid should fully cover the borders of the filter insert.
     4. Incubate at 37 °C, 5% CO_2_ for two days. Repeat steps (i) to (iii) every other day for the desired period of treatment. A treatment length of 2 to 6 days is recommended. Monitor sprout growth regularly using a bright-field microscope.

**Fixation TIMING ~1 d**

1. Fixation of the fibrin gels and subsequent staining. Use option A for 96-well plates, staining for nuclei and cytoskeleton. Use option B for glass-bottom dishes to perform immunofluorescence staining.
   1. **Fixation of gels in 96-well plate format TIMING ~1 d**
      1. Remove growth media from the fibrin gels by carefully inverting the plate to rinse the supernatant, followed by dipping the plate onto tissue paper to remove most of the liquid.
      2. Wash the plate by filling a plastic tray with DPBS and gently submerging the plate to fill each well. Keep on RT for 5 min.
      3. Wash a second time by repeating steps (i) and (ii).
      4. Remove supernatant by inverting the plate and dipping on tissue paper. Using a multi-channel pipet, transfer 200 µl 4% paraformaldehyde (PFA) in DPBS into each well.
      5. Keep the plate overnight at 4 °C.
      6. Wash the plate twice with DPBS as in step (ii).
   2. **Removal of filter insert and fixation of gels in a glass-bottom culture dish TIMING ~4 h**
      1. Carefully remove the filter insert by lifting it with a forceps while the gel remains immersed in growth medium.

**CRITICAL STEP** The main part of the fibrin gel will stick to the filter insert. While carefully removing the insert, make sure that the microcarrier beads and sprouts remain attached to the glass bottom of the culture dish.

- - 1. Wash the gel 3 times by aspirating the supernatant, adding 3 ml DPBS and keeping the gel at room temperature for 5 min.
    2. Aspirate and discard the supernatant.
    3. Add 3 ml of a 4% PFA solution into the culture dish and incubate for 2 h at room temperature.
    4. Wash 3 times with DPBS.

**PAUSE POINT** The protocol may be interrupted at this point: after fixation, gels can be stored for up to four weeks when covered with a sufficient amount of DPBS to prevent them from drying out.

**Blocking and Staining TIMING variable**

1. Staining of the vascular sprouts can be performed in three ways. A one-step staining protocol is recommended for EC sprouts in 96-well plate format, by following option A (Phalloidin-AF488, DAPI), whereas a two-step protocol is used for immunostaining of sprouts containing both pericytes and ECs (option B). When grown in glass-bottom dishes, sprouts can be stained using a shorter two-step immunostaining protocol (option C).
   1. **One-step staining for F-actin and nuclei (96-well format) TIMING 3 d**
      1. Remove the supernatant of the wells by inverting the plate and dipping it carefully on tissue paper.
      2. Add 200 µl 0.2% Triton blocking solution per well using a multi-channel pipette.
      3. Incubate for 6 h or overnight at room temperature.
      4. Prepare the staining solution (3.5 ml per plate) by diluting Alexa Fluor 488 phalloidin 1:500, DAPI 1:1000 in 0.2% Triton blocking solution.
      5. Remove the supernatant by inverting the plate and dipping it on tissue paper.
      6. Add 50 µl staining solution per well using a multi-channel pipette.
      7. Incubate for at least 40 h at room temperature with gentle agitation.
      8. Remove the supernatant by inverting the plate and dipping it on tissue paper.
      9. Wash three times with 200 µl DPBS per well and incubate the plate during each washing step for 10 min at room temperature.
      10. Check the staining quality by inspecting the plate with an epifluorescence microscope.
   2. **Two-step immunostaining (96-well plate format) TIMING 10 d**
      1. Remove the supernatant of the wells by inverting the plate and dipping it carefully on tissue paper.
      2. Add 200 µl 3% Triton blocking solution per well using a multi-channel pipette.
      3. Wrap the plate in laboratory film to prevent loss of liquid, and incubate 15 min at 37°C with gentle agitation.
      4. Incubate overnight at 37°C.
      5. Prepare the primary staining solution (2 ml per plate) by diluting the primary antibodies (e.g. rabbit anti-human Erg-1/2/3, 1:50; and mouse anti-human NG2, 1:200) in 3% Triton blocking solution.
      6. Remove the laboratory film from the plate and discard the supernatant by inverting the plate and dipping it on tissue paper.
      7. Add 30 µl primary staining solution per well using a multi-channel pipette.
      8. Wrap the plate in laboratory film and incubate at 37°C for at least 60 h.
      9. Remove the laboratory film from the plate and discard the supernatant by inverting the plate and dipping it on tissue paper.
      10. Wash with 3% Triton X-100 in DPBS (washing solution) by incubating at 37°C for at least 40 h and changing the washing solution at least three times during this period.
      11. Prepare the secondary staining solution (2 ml per plate) by diluting the secondary antibodies (e.g. Alexa Flour 488 donkey anti-rabbit IgG, 1:500; and Cy3 donkey anti-mouse IgG, 1:500), Alexa Fluor 647 phalloidin (1:500), and DAPI (1:1000) in DPBS containing 3% Triton X-100.
      12. Discard the supernatant from the plate by inverting the plate and dipping it on tissue paper.
      13. Add 30 µl secondary staining solution per well using a multi-channel pipette.
      14. Wrap the plate in laboratory film and incubate at 37°C for at least 40 h.
      15. Remove the laboratory film from the plate and discard the supernatant by inverting the plate and dipping it on tissue paper.
      16. Wash with DPBS by incubating at 37°C for at least 40 h and changing the DPBS solution at least three times during this period.
      17. Check the staining quality by inspecting the plate with an epifluorescence microscope.
   3. **Two-step immunostaining (glass-bottom culture dish format) TIMING 1 d**
      1. Aspirate and discard the supernatant. Carefully tilt the culture dish and aspirate from the border of the dish with a thin Pasteur pipet connected to a vacuum pump.
      2. Add 1 ml 0.2% Triton blocking solution and incubate for at least 2 h at RT while gently agitating on a laboratory shaker.
      3. Prepare primary antibody staining solution by diluting the desired primary antibodies (e.g. mouse anti-VE-cadherin, 1:100; rabbit anti-Laminin, 1:100) in 0.2% Triton blocking solution.
      4. Aspirate the supernatant and carefully distribute 50 µl primary antibody solution onto the fibrin gel and incubate overnight at 4 °C in a humid chamber.
      5. Wash 3 times for 5 min with DPBS.
      6. Prepare secondary antibody solutions by diluting the appropriate secondary antibodies (e.g. Alexa Fluor 488 donkey anti-mouse IgG, 1:500; and Alexa Fluor 647 donkey anti-rabbit IgG, 1:500) and DAPI (1:1000) in 0.2% Triton blocking solution.
      7. Aspirate the supernatant and carefully distribute 100 µl secondary antibody solution onto the fibrin gel and incubate at room temperature for 2 hours.
      8. Wash 3 times for 5 min with DPBS.
      9. Mount the gel by covering with mounting solution and carefully placing a cover glass onto the gel, using forceps.
      10. Check the staining quality by inspecting the sample with an epifluorescence microscope.

**Imaging TIMING variable, ~1 d per plate or 20-40 min for single 3D stacks**

1. For imaging of multi-well plates using an automated microscope, follow option A. For imaging immunostained sprouts on a confocal laser scanning microscope, follow option B.
   1. **Multi well tile scan (96-well plate format) TIMING variable, 4-12 h**
      1. Position the plate in the holding frame of the microscope and, if necessary, calibrate the table positioning using the microscope software.
      2. Using a multi-position tile scan, acquire images covering a large field of view within each well. For a full plate with 60 wells, this will result in a dataset of, e.g. 60 positions, 4 x 5 tiles, 2 channels, 15 slices.
      3. Save the image files in a format that preserves metadata, e.g. .czi or .lsm, or export them in OME-XML format.
   2. **High magnification laser scanning confocal microscopy TIMING 20-40 min**
      1. Acquire z-stack images of single beads with sprouts or of single sprouts using a laser scanning confocal microscope.
      2. Save the image files in a format that preserves metadata. On a Zeiss LSM, save the image as .lsm file containing all positions.

**Image analysis TIMING variable, ~2-20 h**

Images of sprouts can be analyzed using the plugin we developed (see also movies **Additional Files 1 and 2**). For morphometrical analysis of sprouts, maximum intensity projection (MIP) images need to be created from the 3-dimensional image data that then can be measured using our Fiji plugin.

1. Prepare MIP images using Fiji. Open every image dataset and perform a maximum intensity z projection (using *Image > Stacks > Z project…* in the menu or *run(“Z project…”, “projection=[Max Intensity]”)* as macro command). To convert a whole folder of images into MIP, we provide a Fiji script (see **Additional File 3**).

**Plugin configuration with representative images TIMING variable, 0.5-1 h**

1. Open the MIP of a representative image and start the “Sprout Analysis” plugin by running *Analyze > Sprout Morphology* in the menu. A series of dialogs allow the choice of analysis parameters (**Fig. 3** and **Additional Files 1 and 2**).

**CRITICAL STEP** The choice of representative sample images to configure the analysis is critical for getting a robust quantification.

1. In the first configuration dialog, choose the desired measurement parameters and the channels of your image containing the respective staining. We recommend measuring bead number, sprout number, sprout length, sprout width, and cell density for EC sprouts, and in addition numbers of EC/pericytes and pericyte coverage for sprouts where pericytes are present. For the detection of beads, select the channel containing DAPI staining, as the microcarrier beads get stained by DAPI (**Fig. 2a-c**).
2. The second dialog configures the bead segmentation (**Fig. 2d-f**). The set of default values can be adjusted to optimize bead detection. A preview option is available to allow refinement of parameters.
3. The third dialog configures sprout detection (**Fig. 2g-i**). The default values can be adjusted and the resulting segmentation can be controlled using the preview function.
4. The nucleus segmentation dialog (**Fig. 2j**) only appears if a cell-related parameter was chosen in the first configuration dialog. Using the preview function, adjust the default values if necessary to detect and separate single nuclei (**Fig. 2k** and **2l**).
5. The cell classification (**Fig. 2m**) and pericyte coverage dialogs (**Fig. 2o**) are optional and appear only if a pericyte-related parameter was chosen in the first configuration dialog. Using the preview function, cell classification and pericyte area detection can be adjusted (**Fig. 2n** and **2p**). After pressing *OK* in the last dialog, a new window opens showing the segmentation result as white masks on black background. In the results table, a row is added for each analyzed image.
6. Using several representative images (from Step 26), find a suitable set of analysis parameters by opening one image at a time, running the Sprout Morphology plugin, and adjusting the parameters if necessary. The parameters will be saved upon running the analysis, so they can be re-used on another image.

**Image analysis using the configured plugin**

1. Once a suitable set of parameters has been found, start the macro recorder of ImageJ (*Plugins > Macros > Record…*), and run the plugin once more to record the necessary command for sprout analysis. Copy and paste the command into the analysis script provided in **Additional File 3**.
2. To run the analysis on a directory containing multiple images, run the analysis script (see script in **Additional File 3**) and choose the respective directory. The results will be saved into a .xls file that can be opened with any spreadsheet software, e.g. Microsoft Excel, or imported into a statistical software.
3. Investigate the resulting images and control their quality by comparing representative result images with the corresponding original images.

**Statistical analysis**

1. Open the results file in statistics software of your choice. For all replicated conditions within the same plate, calculate the means by averaging all respective replicates. If necessary, the calculated means can be weighted according to the number of beads in each well. Normalize to the intra-plate controls by dividing the means of each condition by the mean of the suitable control condition.
