# Supplementary material for: Quantitative assessment of angiogenesis and pericyte coverage in human cell-derived vascular sprouts
Source: Inflamm Regen. 2017 Jan 18;37:2. doi: 10.1186/s41232-016-0033-2 (PMC5725907; doi:10.1186/s41232-016-0033-2)
Supplement: Supplementary file 6 — List of compounds and concentrations used. The compounds were used at two concentrations, indicated in the columns 10 × IC50 and 100 × IC50, respectively. The values were chosen according to published values for the half maximal inhibitory concentrations of the inhibitors with respect to their target molecules in vitro or in accordance with literature using the respective compounds in a similar assay setup. Literature references for each compound are listed in the reference column. (DOCX 193 kb) [file 41232_2016_33_MOESM6_ESM.docx]

| **Compound Abbreviation** | **Compound Name** | **10 × IC_50_**^[[1]](#footnote-1)^ | **100 × IC_50_**^a^ | **Solvent** | **Ref** |
| --- | --- | --- | --- | --- | --- |
| 6-Bnz-cAMP | N6- Benzoyladenosine- 3', 5'- cyclic monophosphate | 22 µM | 0.22 mM | H_2_O | [39] |
| Blebbistatin | (±)-1,2,3,3a-Tetrahydro-3a-hydroxy-6  -methyl-1-phenyl-4H-pyrrolo[2,3-b]quinolin-4-one | 30 µM | 0.3 mM | DMSO | [40-42] |
| Calpeptin | N-Benzyloxycarbonyl-L-leucyl-norleucinal | 1 µM | 10 µM | DMSO | [43] |
| Calphostin C | Calphostin C | 0.5 µM | 5 µM | DMSO | [44] |
| Cilostamide | 6-[3-(N-Cyclohexyl-N-methylcarbamoyl)propoxy]quinolin-2[1H]-one | 1 µM | 10 µM | DMSO | [45] |
| Cyclopamine | (3b,23b)-17,23-Epoxy-11-deoxoveratraman-3-ol | 0.24 µM | 2.4 µM | DMSO | [46] |
| Cytochalasin D | Cytochalasin D | 1 µM | 10 µM | DMSO | [47, 42] |
| DAPT | N-[N-(3,5-Difluorophenacetyl)-L-alanyl]-S-phenylglycine t-butyl ester | 1 µM | 10 µM | DMSO | [48] |
| DDA | Dideoxyadenosine | 1 µM | 10 µM | DMSO | [49] |
| EHNA | Erythro-9-(2-hydroxy-3-nonyl)adenine | 8 µM | 80 µM | H_2_O | [50, 51] |
| Eph-inh 6 | N-(2-methyl-5-(3-(4-methyl-1H-imidazol-1yl)-5(trifluoro­methyl)benzamido)phenyl)isoxazole-5- carboxamide | 0.1 µM | 1 µM | DMSO | [52] |
| Eph-inh 9 | 5-(2-methyl-5-(3-(trifluoromethyl)phenylcarbamoyl)­phenylamino)nicotinamide | 0.1 µM | 1 µM | DMSO | [52] |
| Forskolin | 7β-Acetoxy-8,13-epoxy-1α,6β,9α-trihydroxylabd-14-en-11-one | 10 µM^*^ | 0.1 mM | DMSO | [53] |
| GM6001 | N-[(2R)-2-(Hydroxamidocarbonylmethyl)-4-methylpentanoyl]-L-tryptophan Methylamide | 5 nM | 50 nM | DMSO | [54] |
| Gö-6976 | 12-(2-Cyanoethyl)-6,7,12,13-tetrahydro-13-methyl-5-oxo-5H-indolo[2,3-a]pyrrolo[3,4-c]carbazole | 0.1 µM | 1 µM | DMSO | [55, 56] |
| Gö-6983 | 3-[1-[3-(Dimethylamino)propyl]-5-methoxy-1H-indol-3-yl]-4-(1H-indol-3-yl)-1H-pyrrole-2,5-dione | 0.5 µM | 5 µM | DMSO | [57, 58] |
| GSK3β inh | GSK-3β inhibitor IX, (2′Z,3′E)-6-Bromoindirubin-3′-oxime | 50 nM | 0.5 µM | DMSO | [59] |
| H-89 | N-[2-[[3-(4-Bromophenyl)-2-propenyl  ]amino]ethyl]-5-isoquinolinesulfonamide dihydrochloride | 0.5 µM | 5 µM | H_2_O | [60] |
| H1152 | (S)-(+)-2-Methyl-1-[(4-methyl-5-iso  quinolinyl)sulfonyl]-hexahydro-1H-1,4-diazepine dihydrochloride | 0.5 µM | 5 µM | H_2_O | [61, 62] |
| IBMX | 3-Isobutyl-1-methylxanthine | 20 µM | 0.2 mM | DMSO | [63] |
| Jasplakinolide | Jasplakinolide | 1 µM | 10 µM | DMSO | [64, 42] |

| **Compound Abbreviation** | **Compound Name** | **10 × IC_50_**^a^ | **100 × IC_50_**^a^ | **Solvent** | **Ref** |
| --- | --- | --- | --- | --- | --- |
| L-NOARG | L-NG-nitro arginine | 14 µM | 0.14 mM | H_2_O | [65, 66] |
| Latrunculin B | Latrunculin B | 0.3 µM | 3 µM | DMSO | [67, 42] |
| ML-7 | 1-(5-Iodonaphthalene-1-sulfonyl)-1H-hexahydro-1,4-diazepine hydrochloride | 3 µM | 30 µM | DMSO | [68] |
| NFκB inh | 6-Amino-4-(4-phenoxyphenylethylamino)quinazoline | 0.11 µM | 1.1 µM | DMSO | [69] |
| NKH477 | 6-(3-Dimethylaminopropionyl)forskolin | 0.7 µM | 7 µM | H_2_O | [70] |
| Nocodazole | Methyl N-(5-thenoyl-2-benzimidazolyl)carbamate | 10 µM | 0.1 mM | DMSO | [71, 72] |
| PMA | Phorbol 12-myristate 13-acetate | 80 nM | 0.8 µM | DMSO | [73, 74] |
| PP2 | 3-(4-chlorophenyl) 1-(1,1-dimethylethyl)-1H-pyrazolo[3,4-d]pyrimidin-4-amine | 50 nM | 0.5 µM | DMSO | [75] |
| Ro31-8220 | 2-[1-[3-(Amidinothio)propyl]-1H-indol-3-yl]-3-(1-methylindol-3-yl)maleimide methanesulfonate salt | 1 µM | 10 µM | DMSO | [76] |
| Rolipram | 4-[3-(Cyclopentyloxy)-4-methoxyphenyl]-2-pyrrolidinone | 3 µM | 30 µM | DMSO | [77] |
| SNAP | S-nitroso-N-acetylpenicillamine | 10 µM | 0.1 mM | DMSO | [78] |
| Sp-cAMPS | (S)-Adenosine, cyclic 3',5'-(hydrogenphosphorothioate) triethylammonium | 20 µM | 0.2 mM | H_2_O | [79] |
| SQ22536 | 9-(Tetrahydro-2-furanyl)-9H-purin-6-amine | 0.2 mM | 2 mM | DMSO | [80, 81] |
| SU5416 | (3Z)-3-[(3,5-dimethyl-1H-pyrrol-2-yl)methylidene]-1,3-dihydro-2H-indol-2-one | 0.4 µM | 4 µM | DMSO | [82] |
| TAPI-1 | N-(R)-[2-(Hydroxyaminocarbonyl)methyl]-4-methylpentanoyl-L-naphthylalanyl-L-alanine | 10 µM | 0.1 mM | DMSO | [83] |
| Taxol | Paclitaxel | 0.11 µM | 1.1 µM | DMSO | [84] |
| TGFβRI inh. | [3-(Pyridin-2-yl)-4-(4-quinonyl)]-1H-pyrazole | 0.5 µM | 5 µM | DMSO | [85] |
| Vanadate | Orthovanadate | 10 µM | 0.1 mM | H_2_O | [86, 87] |
| Y27632 | (R)-(+)-trans-4-(1-Aminoethyl)-N-(4-Pyridyl)cyclo­hexanecarboxamide dihydrochloride monohydrate | 1 µM | 10 µM | H_2_O | [71] |
| γ-sec. inh. X | {1S-Benzyl-4R-[1-(1S-carbamoyl-2-phenethyl­carbamoyl)-1S-3-methylbutylcarbamoyl]-2R-hydroxy-5-phenylpentyl}carbamic Acid *tert*-butyl Ester | 0.1 µM | 1 µM | DMSO | [88] |
| (*only in EC/pericyte assay*) |  |  |  |  |  |
| PDGFR inh. IV | 3-Fluoro-N-(6,7-dimethoxy-2,4-dihydroindeno[1,2-c]pyrazol-3-yl)phenylamine | 0.4 µM | 4 µM | DMSO | [89] |

References

39. Hewer RC, Sala-Newby GB, Wu YJ, Newby AC, Bond M. PKA and Epac synergistically inhibit smooth muscle cell proliferation. Journal of Molecular and Cellular Cardiology. 2011;50:87-98. doi:10.1016/j.yjmcc.2010.10.010.

40. Limouze J, Straight AF, Mitchison T, Sellers JR. Specificity of blebbistatin, an inhibitor of myosin II. Journal of Muscle Research and Cell Motility. 2004;25:337-41. doi:10.1007/s10974-004-6060-7.

41. Nakayama M, Amano M, Katsumi A, Kaneko T, Kawabata S, Takefuji M et al. Rho-kinase and myosin II activities are required for cell type and environment specific migration. Genes to Cells. 2005;10:107-17. doi:10.1111/j.1365-2443.2005.00823.x.

42. Peng GE, Wilson SR, Weiner OD. A pharmacological cocktail for arresting actin dynamics in living cells. Molecular Biology of the Cell. 2011;22:3986-94. doi:10.1091/mbc.E11-04-0379.

43. Tabata C, Tabata R, Nakano T. The calpain inhibitor calpeptin prevents bleomycin-induced pulmonary fibrosis in mice. Clinical and Experimental Immunology. 2010;162:560-7. doi:10.1111/j.1365-2249.2010.04257.x.

44. Rahman A, Anwar KN, Uddin S, Xu N, Ye RD, Platanias LC et al. Protein Kinase C-delta Regulates Thrombin-Induced ICAM-1 Gene Expression in Endothelial Cells via Activation of p38 Mitogen-Activated Protein Kinase. 2001;21:5554-65. doi:10.1128/MCB.21.16.5554.

45. Takuma K, Lee E, Enomoto R, Mori K, Baba a, Matsuda T. Ibudilast attenuates astrocyte apoptosis via cyclic GMP signalling pathway in an in vitro reperfusion model. British journal of pharmacology. 2001;133:841-8. doi:10.1038/sj.bjp.0704146.

46. Zeng X, Goetz Ja, Suber LM, Scott WJ, Schreiner CM, Robbins DJ. A freely diffusible form of Sonic hedgehog mediates long-range signalling. Nature. 2001;411:716-20. doi:10.1038/35079648.

47. Fronczak JA, Finer JR, Gehler S, Vogt A, Hulkower KI. A Robust 384 ‐ Well Cell Migration Assay for High Content Analysis. Cell.53711-.

48. Yang Y, Duan W, Liang Z, Yi W, Yan J, Wang N et al. Curcumin attenuates endothelial cell oxidative stress injury through Notch signaling inhibition. Cellular Signalling. 2013;25:615-29. doi:10.1016/j.cellsig.2012.11.025.

49. Molinari C, Uberti F, Grossini E, Vacca G, Carda S, Invernizzi M et al. 1alpha,25-dihydroxycholecalciferol induces nitric oxide production in cultured endothelial cells. Cell Physiol Biochem. 2011;27(6):661-8. doi:10.1159/000330075.

50. Favot L, Keravis T, Holl V, Le Bec A, Lugnier C. VEGF-induced HUVEC migration and proliferation are decreased by PDE2 and PDE4 inhibitors. Thrombosis and Haemostasis. 2003;90:334-43. doi:10.1160/TH03-02-0084.

51. Wang H, Yoshizumi M, Lai K, Tsai J-c, Perrella MA, Haber E et al. Inhibition of Growth and p21 ras Methylation in Vascular Endothelial Cells by Homocysteine but Not Cysteine *. October. 1997;272:25380 -5.

52. Choi Y, Syeda F, Walker JR, Finerty PJ, Cuerrier D, Wojciechowski A et al. Discovery and structural analysis of Eph receptor tyrosine kinase inhibitors. Bioorganic & medicinal chemistry letters. 2009;19:4467-70. doi:10.1016/j.bmcl.2009.05.029.

53. Namkoong S, Kim CK, Cho YL, Kim JH, Lee H, Ha KS et al. Forskolin increases angiogenesis through the coordinated cross-talk of PKA-dependent VEGF expression and Epac-mediated PI3K/Akt/eNOS signaling. Cellular Signalling. 2009;21:906-15. doi:10.1016/j.cellsig.2009.01.038.

54. Elhabazi A, Delaire S, Bensussan A, Boumsell L, Bismuth G. Biological Activity of Soluble CD100. I. The Extracellular Region of CD100 Is Released from the Surface of T Lymphocytes by Regulated Proteolysis. The Journal of Immunology. 2001;166:4341-7. doi:10.4049/jimmunol.166.7.4341.

55. Holinstat M, Mehta D, Kozasa T, Minshall RD, Malik AB. Protein kinase Cα-induced p115RhoGEF phosphorylation signals endothelial cytoskeletal rearrangement. Journal of Biological Chemistry. 2003;278:28793-8. doi:10.1074/jbc.M303900200.

56. Behrens MM, Strasser U, Choi DW. Go 6976 is a potent inhibitor of neurotrophin-receptor intrinsic tyrosine kinase. Journal of Neurochemistry. 1999;72:919-24. doi:10.1046/j.1471-4159.1999.0720919.x.

57. Quann EJ, Liu X, Altan-Bonnet G, Huse M. A cascade of protein kinase C isozymes promotes cytoskeletal polarization in T cells. Nature immunology. 2011;12:647-54. doi:10.1038/ni.2033.

58. De Lichtervelde L, Antal CE, Boitano AE, Wang Y, Krastel P, Petersen F et al. Euphohelioscopin A is a PKC activator capable of inducing macrophage differentiation. Chemistry and Biology. 2012;19:994-1000. doi:10.1016/j.chembiol.2012.06.010.

59. Kohler EE, Baruah J, Urao N, Ushio-Fukai M, Fukai T, Chatterjee I et al. Low-dose 6-bromoindirubin-3′-oxime induces partial dedifferentiation of endothelial cells to promote increased neovascularization. Stem Cells. 2014;32:1538-52. doi:10.1002/stem.1658.

60. Fukuhara S, Sakurai A, Sano H, Somekawa S, Takakura N, Yamagishi A et al. Cyclic AMP Potentiates Vascular Contact To Enhance Endothelial Barrier Function through an Epac-Rap1 Signaling Pathway Cyclic AMP Potentiates Vascular Endothelial Cadherin-Mediated Cell-Cell Contact To Enhance Endothelial Barrier Function through an Epac-. Society. 2005;25:136-46. doi:10.1128/MCB.25.1.136.

61. Breyer J, Samarin J, Rehm M, Lautscham L, Fabry B, Goppelt-Struebe M. Inhibition of Rho kinases increases directional motility of microvascular endothelial cells. Biochemical Pharmacology. 2012;83:616-26. doi:10.1016/j.bcp.2011.12.012.

62. Brown M, Roulson J-a, Hart Ca, Tawadros T, Clarke NW. Arachidonic acid induction of Rho-mediated transendothelial migration in prostate cancer. British journal of cancer. 2014;110:2099-108. doi:10.1038/bjc.2014.99.

63. Griffith TM, Chaytor AT, Taylor HJ, Giddings BD, Edwards DH. cAMP facilitates EDHF-type relaxations in conduit arteries by enhancing electrotonic conduction via gap junctions. Proceedings of the National Academy of Sciences of the United States of America. 2002;99:6392-7. doi:10.1073/pnas.092089799.

64. Cramer LP. Role of actin-filament disassembly in lamellipodium protrusion in motile cells revealed using the drug jasplakinolide. Current Biology. 1999;9:1095-105. doi:10.1016/S0960-9822(99)80478-3.

65. Law a, Gauthier S, Quirion R. Neuroprotective and neurorescuing effects of isoform-specific nitric oxide synthase inhibitors, nitric oxide scavenger, and antioxidant against beta-amyloid toxicity. British journal of pharmacology. 2001;133:1114-24. doi:10.1038/sj.bjp.0704179.

66. Moore PK, al-Swayeh OA, Chong NW, Evans RA, Gibson A. L-NG-nitro arginine (L-NOARG), a novel, L-arginine-reversible inhibitor of endothelium-dependent vasodilatation in vitro. British journal of pharmacology. 1990;99:408-12.

67. Wakatsuki T, Schwab B, Thompson NC, Elson EL. Effects of cytochalasin D and latrunculin B on mechanical properties of cells. Journal of cell science. 2001;114:1025-36.

68. Stroka KM, Aranda-Espinoza H. Endothelial cell substrate stiffness influences neutrophil transmigration via myosin light chain kinase-dependent cell contraction. Blood. 2011;118(6):1632-40. doi:10.1182/blood-2010-11-321125.

69. Tobe M, Isobe Y, Tomizawa H, Nagasaki T, Takahashi H, Hayashi H. A novel structural class of potent inhibitors of NF-kappa B activation: structure-activity relationships and biological effects of 6-aminoquinazoline derivatives. Bioorganic & medicinal chemistry. 2003;11:3869-78. doi:10.1016/S0968-0896(03)00438-3.

70. Satake K, Takagi K, Kodama I, Honjo H, Toyama J, Shibata S. Relaxant effects of NKH477, a new water-soluble forskolin derivative, on guinea-pig tracheal smooth muscle: the role of Ca2+-activated K+ channels. Br J Pharmacol. 1998;123(4):753-61. doi:10.1038/sj.bjp.0701655.

71. Tee YH, Shemesh T, Thiagarajan V, Hariadi RF, Anderson KL, Page C et al. Cellular chirality arising from the self-organization of the actin cytoskeleton. Nature cell biology. 2015;17:445-57. doi:10.1038/ncb3137.

72. Blajeski AL, Phan Va, Kottke TJ, Kaufmann SH. G 1 and G 2 cell-cycle arrest following microtubule depolymerization in human breast cancer cells. The Journal of Clinical Investigation. 2002;110:91-9. doi:10.1172/JCI200213275.Introduction.

73. Xu YC, Leung GPH, Wong PYD, Vanhoutte PM, Man RYK. Kaempferol stimulates large conductance Ca2+ -activated K+ (BKCa) channels in human umbilical vein endothelial cells via a cAMP/PKA-dependent pathway. British journal of pharmacology. 2008;154:1247-53. doi:10.1038/bjp.2008.194.

74. Langer DJ, Kuo A, Kariko K, Ahuja M, Klugherz BD, Ivanics KM et al. Regulation of the endothelial cell urokinase-type plasminogen activator receptor. Evidence for cyclic AMP-dependent and protein kinase C-dependent pathways. CircRes. 1993;72:330-40.

75. Nam J-s, Ino Y, Sakamoto M, Hirohashi S. Src family kinase inhibitor PP2 restores the E-cadherin/catenin cell adhesion system in human cancer cells and reduces cancer metastasis. Clinical Cancer Research. 2002;8:2430-6. doi:do.

76. Muller G, Catar RA, Niemann B, Barton M, Knels L, Wendel M et al. Upregulation of endothelin receptor B in human endothelial cells by low-density lipoproteins. Experimental biology and medicine (Maywood, NJ). 2006;231:766-71.

77. Barad M, Bourtchouladze R, Winder DG, Golan H, Kandel E. Rolipram, a type IV-specific phosphodiesterase inhibitor, facilitates the establishment of long-lasting long-term potentiation and improves memory. Proceedings of the National Academy of Sciences of the United States of America. 1998;95:15020-5. doi:10.1073/pnas.95.25.15020.

78. Collard CD, Agah A, Reenstra W, Buras J, Stahl GL. Endothelial nuclear factor-kappaB translocation and vascular cell adhesion molecule-1 induction by complement: inhibition with anti-human C5 therapy or cGMP analogues. Arteriosclerosis, thrombosis, and vascular biology. 1999;19:2623-9. doi:10.1161/01.ATV.19.11.2623.

79. Ming GL, Song HJ, Berninger B, Holt CE, Tessier-Lavigne M, Poo MM. cAMP-dependent growth cone guidance by netrin-1. Neuron. 1997;19:1225-35. doi:10.1016/S0896-6273(00)80414-6.

80. Rhim JH, Jang IS, Kwon ST, Song KY, Yeo EJ, Park SC. Activation of wound healing in aged rats by altering the cellular mitogenic potential. Journals of Gerontology - Series A Biological Sciences and Medical Sciences. 2010;65 A:704-11. doi:10.1093/gerona/glq065.

81. Xu H, Czerwinski P, Hortmann M, Sohn H-Y, Förstermann U, Li H. Protein kinase C alpha promotes angiogenic activity of human endothelial cells via induction of vascular endothelial growth factor. Cardiovascular research. 2008;78:349-55. doi:10.1093/cvr/cvm085.

82. Mendel DB, Schreck RE, West DC, Li G, Strawn LM, Tanciongco SS et al. The angiogenesis inhibitor SU5416 has long-lasting effects on vascular endothelial growth factor receptor phosphorylation and function. Clinical Cancer Researchpharmacological. 2000;6:4848-58.

83. Fröhlich C, Klitgaard M, Noer JB, Kotzsch A, Nehammer C, Kronqvist P et al. ADAM12 is expressed in the tumour vasculature and mediates ectodomain shedding of several membrane-anchored endothelial proteins. The Biochemical journal. 2013;452:97-109. doi:10.1042/BJ20121558.

84. Kitamura N, Sakamoto K, Ono T, Kimura J. THE INHIBITORY EFFECT OF PACLITAXEL ON (KV2.1) K(+) CURRENT IN H9c2 CELLS. Fukushima journal of medical science. 2015;61:47-53. doi:10.5387/fms.2014-34.

85. Sawyer JS, Anderson BD, Beight DW, Campbell RM, Jones ML, Herron DK et al. Synthesis and activity of new aryl- and heteroaryl-substituted pyrazole inhibitors of the transforming growth factor-β type I receptor kinase domain. Journal of Medicinal Chemistry. 2003;46:3953-6. doi:10.1021/jm0205705.

86. Morita a, Zhu J, Suzuki N, Enomoto a, Matsumoto Y, Tomita M et al. Sodium orthovanadate suppresses DNA damage-induced caspase activation and apoptosis by inactivating p53. Cell death and differentiation. 2006;13:499-511. doi:10.1038/sj.cdd.4401768.

87. Sakai A. Orthovanadate , an inhibitor of protein tyrosine phosphatases , acts more potently as a promoter than as an initiator in the BALB / 3T3 cell transformation. 1997;18:1395-9.

88. Schulz B, Pruessmeyer J, Maretzky T, Ludwig A, Blobel CP, Saftig P et al. ADAM10 regulates endothelial permeability and T-Cell transmigration by proteolysis of vascular endothelial cadherin. Circ Res. 2008;102(10):1192-201. doi:10.1161/CIRCRESAHA.107.169805.

89. Ball SG, Shuttleworth A, Kielty CM. Inhibition of platelet-derived growth factor receptor signaling regulates Oct4 and Nanog expression, cell shape, and mesenchymal stem cell potency. Stem Cells. 2012;30(3):548-60. doi:10.1002/stem.1015.

1. Where no IC_50_ value was available, concentrations were based on those used in literature. [↑](#footnote-ref-1)
