# Supplementary material for: Quantitative assessment of angiogenesis and pericyte coverage in human cell-derived vascular sprouts
Source: Inflamm Regen. 2017 Jan 18;37:2. doi: 10.1186/s41232-016-0033-2 (PMC5725907; doi:10.1186/s41232-016-0033-2)
Supplement: Supplementary file 7 — Results of a pilot screen for EC-derived vascular sprouts. Changes considered significant with a cut-off p value of p < 0.05 (two-sided one-sample t test compared to solvent control) are colored in red and green to indicate decrease and increase compared to controls, respectively. N = 3 assays with 8 wells per compound each. (PDF 66 kb) [file 41232_2016_33_MOESM7_ESM.pdf]

Results from a pilot screen for EC sprouts

**Condition**	**Number of sprouts** (relative to control)	**Sprout length** (relative to control)	**Sprout width** (relative to control)	**Cell density** (relative to control)
6-Bnz 10×	0.98 ± 0.31 (p=0.915)	0.85 ± 0.24 (p=0.407)	1.21 ± 0.27 (p=0.315)	1.14 ± 0.28 (p=0.481)
6-Bnz 100×	1.30 ± 0.14 (p=0.064)	1.02 ± 0.53 (p=0.962)	1.10 ± 0.13 (p=0.296)	0.94 ± 0.27 (p=0.755)
Blebbistatin 10×	1.40 ± 0.53 (p=0.326)	1.03 ± 0.24 (p=0.857)	0.96 ± 0.07 (p=0.435)	0.86 ± 0.12 (p=0.185)
Blebbistatin 100×	1.79 ± 0.54 (p=0.128)	1.02 ± 0.13 (p=0.826)	0.91 ± 0.08 (p=0.185)	0.85 ± 0.13 (p=0.194)
Calpeptin 10×	1.14 ± 0.25 (p=0.442)	1.06 ± 0.36 (p=0.792)	0.91 ± 0.11 (p=0.297)	1.05 ± 0.29 (p=0.789)
Calpeptin 100×	0.94 ± 0.33 (p=0.779)	0.85 ± 0.22 (p=0.343)	1.33 ± 0.48 (p=0.358)	0.99 ± 0.01 (p=0.134)
Calphostin C 10×	1.15 ± 0.26 (p=0.431)	0.91 ± 0.05 (p=0.091)	0.91 ± 0.01 (p=0.004)	0.85 ± 0.03 (p=0.017)
Calphostin C 100×	1.07 ± 0.12 (p=0.392)	0.80 ± 0.51 (p=0.577)	1.02 ± 0.18 (p=0.883)	0.73 ± 0.23 (p=0.176)
Cilostamide 10×	1.54 ± 1.34 (p=0.555)	1.01 ± 0.11 (p=0.882)	1.06 ± 0.03 (p=0.093)	1.00 ± 0.11 (p=0.998)
Cilostamide 100×	0.82 ± 0.51 (p=0.605)	0.68 ± 0.43 (p=0.323)	0.96 ± 0.04 (p=0.248)	0.89 ± 0.14 (p=0.322)
Cyclopamine 10×	0.90 ± 0.41 (p=0.713)	0.76 ± 0.17 (p=0.126)	1.16 ± 0.23 (p=0.340)	0.98 ± 0.11 (p=0.748)
Cyclopamine 100×	1.01 ± 0.50 (p=0.979)	0.95 ± 0.12 (p=0.578)	1.12 ± 0.13 (p=0.250)	0.82 ± 0.06 (p=0.037)
Cytochalasin D 10×	0.49 ± 0.09 (p=0.009)	0.58 ± 0.18 (p=0.056)	0.84 ± 0.14 (p=0.185)	0.72 ± 0.03 (p=0.004)
Cytochalasin D 100×	0.11 ± 0.10 (p=0.004)	0.32 ± 0.29 (p=0.055)	0.57 ± 0.50 (p=0.275)	0.28 ± 0.46 (p=0.111)
DAPT 10×	0.73 ± 0.37 (p=0.335)	0.88 ± 0.33 (p=0.601)	0.96 ± 0.07 (p=0.421)	1.05 ± 0.04 (p=0.131)
DAPT 100×	0.68 ± 0.29 (p=0.204)	0.84 ± 0.23 (p=0.362)	1.01 ± 0.23 (p=0.964)	0.99 ± 0.19 (p=0.943)
DDA 10×	0.94 ± 0.06 (p=0.201)	0.77 ± 0.23 (p=0.226)	1.90 ± 1.33 (p=0.363)	1.13 ± 0.09 (p=0.126)
DDA 100×	0.86 ± 0.30 (p=0.516)	0.88 ± 0.11 (p=0.196)	1.13 ± 0.23 (p=0.427)	0.99 ± 0.12 (p=0.915)
EHNA 10×	0.63 ± 0.31 (p=0.176)	0.77 ± 0.20 (p=0.180)	1.03 ± 0.21 (p=0.802)	1.14 ± 0.41 (p=0.617)
EHNA 100×	0.47 ± 0.22 (p=0.054)	0.75 ± 0.06 (p=0.019)	0.94 ± 0.02 (p=0.025)	1.23 ± 0.76 (p=0.648)
Eph-inh 6 10×	0.90 ± 0.15 (p=0.385)	1.73 ± 0.85 (p=0.274)	1.11 ± 0.11 (p=0.248)	0.86 ± 0.07 (p=0.078)
Eph-inh 6 100×	0.92 ± 0.31 (p=0.712)	0.96 ± 0.07 (p=0.480)	1.36 ± 0.16 (p=0.058)	1.02 ± 0.11 (p=0.753)
Eph-inh 9 10×	0.98 ± 0.07 (p=0.672)	0.98 ± 0.16 (p=0.871)	1.11 ± 0.04 (p=0.044)	1.03 ± 0.02 (p=0.218)
Eph-inh 9 100×	0.94 ± 0.08 (p=0.362)	0.67 ± 0.02 (p=0.001)	1.19 ± 0.09 (p=0.069)	1.09 ± 0.15 (p=0.403)
Forskolin 10×	1.37 ± 0.01 (p=0.000)	1.23 ± 0.20 (p=0.183)	1.12 ± 0.05 (p=0.060)	0.82 ± 0.13 (p=0.132)
Forskolin 100×	1.41 ± 0.33 (p=0.167)	3.46 ± 4.29 (p=0.424)	1.42 ± 0.68 (p=0.394)	0.69 ± 0.07 (p=0.015)
GM6001 10×	1.09 ± 0.12 (p=0.346)	1.02 ± 0.16 (p=0.882)	0.88 ± 0.04 (p=0.040)	0.76 ± 0.07 (p=0.029)
GM6001 100×	1.16 ± 0.08 (p=0.073)	0.75 ± 0.14 (p=0.090)	1.13 ± 0.09 (p=0.129)	0.95 ± 0.11 (p=0.515)
Gö-6976 10×	0.25 ± 0.23 (p=0.029)	0.83 ± 0.75 (p=0.735)	0.47 ± 0.41 (p=0.158)	0.15 ± 0.13 (p=0.008)
Gö-6976 100×	0.33 ± 0.28 (p=0.054)	0.97 ± 0.14 (p=0.774)	0.76 ± 0.10 (p=0.054)	0.62 ± 0.31 (p=0.161)
Gö-6983 10×	0.61 ± 0.14 (p=0.039)	0.91 ± 0.20 (p=0.503)	0.97 ± 0.20 (p=0.828)	1.16 ± 0.19 (p=0.291)
Gö-6983 100×	0.55 ± 0.28 (p=0.111)	0.80 ± 0.12 (p=0.091)	0.82 ± 0.20 (p=0.251)	0.36 ± 0.15 (p=0.018)
GSK3b inh 10×	0.98 ± 0.12 (p=0.824)	0.81 ± 0.03 (p=0.009)	1.02 ± 0.09 (p=0.791)	0.95 ± 0.12 (p=0.517)
GSK3b inh 100×	0.77 ± 0.09 (p=0.045)	0.85 ± 0.18 (p=0.281)	0.97 ± 0.20 (p=0.839)	0.95 ± 0.11 (p=0.494)
H-89 10×	0.97 ± 0.25 (p=0.852)	0.93 ± 0.15 (p=0.497)	0.99 ± 0.08 (p=0.775)	0.88 ± 0.10 (p=0.169)
H-89 100×	1.35 ± 0.24 (p=0.131)	1.18 ± 0.05 (p=0.026)	0.85 ± 0.11 (p=0.144)	0.74 ± 0.11 (p=0.058)
H1152 10×	1.57 ± 0.21 (p=0.043)	1.16 ± 0.16 (p=0.224)	0.83 ± 0.13 (p=0.154)	0.90 ± 0.26 (p=0.583)
H1152 100×	1.73 ± 0.10 (p=0.006)	1.10 ± 0.19 (p=0.457)	0.80 ± 0.09 (p=0.064)	0.91 ± 0.10 (p=0.270)
IBMX 10×	0.93 ± 0.20 (p=0.580)	1.25 ± 0.73 (p=0.610)	1.15 ± 0.14 (p=0.213)	1.02 ± 0.13 (p=0.793)
IBMX 100×	0.86 ± 0.32 (p=0.518)	0.84 ± 0.14 (p=0.198)	1.06 ± 0.16 (p=0.564)	0.99 ± 0.05 (p=0.791)
Jasplakinolide 10×	0.80 ± 0.31 (p=0.368)	0.74 ± 0.27 (p=0.242)	0.92 ± 0.05 (p=0.104)	1.27 ± 0.15 (p=0.086)
Jasplakinolide 100×	0.55 ± 0.51 (p=0.269)	1.18 ± 1.26 (p=0.829)	1.18 ± 0.21 (p=0.263)	1.01 ± 0.84 (p=0.981)
L-NOARG 10×	1.47 ± 0.26 (p=0.085)	1.05 ± 0.29 (p=0.812)	1.10 ± 0.11 (p=0.238)	0.86 ± 0.17 (p=0.285)
L-NOARG 100×	1.04 ± 0.03 (p=0.116)	1.04 ± 0.16 (p=0.722)	0.92 ± 0.09 (p=0.267)	1.03 ± 0.08 (p=0.550)

Results from a pilot screen for EC sprouts

Condition	Number of sprouts (relative to control)	Sprout length (relative to control)	Sprout width (relative to control)	Cell density (relative to control)
Latrunculin B 10×	1.25 ± 0.36 (p=0.351)	1.14 ± 0.08 (p=0.093)	0.91 ± 0.06 (p=0.125)	0.77 ± 0.31 (p=0.326)
Latrunculin B 100×	0.45 ± 0.03 (p=0.001)	0.61 ± 0.06 (p=0.007)	0.81 ± 0.15 (p=0.170)	1.18 ± 0.23 (p=0.317)
ML-7 10×	1.38 ± 0.07 (p=0.012)	1.13 ± 0.04 (p=0.026)	1.03 ± 0.12 (p=0.663)	1.10 ± 0.26 (p=0.560)
ML-7 100×	0.42 ± 0.12 (p=0.015)	0.71 ± 0.11 (p=0.043)	0.81 ± 0.09 (p=0.066)	1.33 ± 0.14 (p=0.059)
NFkB inh 10×	0.74 ± 0.10 (p=0.047)	0.62 ± 0.15 (p=0.050)	1.03 ± 0.23 (p=0.838)	0.91 ± 0.22 (p=0.551)
NFkB inh 100×	0.66 ± 0.07 (p=0.014)	0.65 ± 0.19 (p=0.083)	1.03 ± 0.19 (p=0.822)	0.85 ± 0.24 (p=0.406)
NKH477 10×	1.44 ± 0.36 (p=0.170)	1.03 ± 0.13 (p=0.725)	1.14 ± 0.10 (p=0.149)	0.88 ± 0.13 (p=0.239)
NKH477 100×	1.83 ± 0.56 (p=0.126)	1.04 ± 0.16 (p=0.733)	1.12 ± 0.17 (p=0.345)	0.74 ± 0.07 (p=0.023)
Nocodazole 10×	0.20 ± 0.10 (p=0.005)	0.31 ± 0.23 (p=0.036)	0.64 ± 0.42 (p=0.270)	0.56 ± 0.35 (p=0.159)
Nocodazole 100×	0.36 ± 0.22 (p=0.036)	0.49 ± 0.19 (p=0.043)	1.14 ± 0.36 (p=0.568)	1.23 ± 0.67 (p=0.616)
PMA 10×	0.74 ± 0.46 (p=0.438)	0.70 ± 0.18 (p=0.100)	0.99 ± 0.07 (p=0.883)	0.66 ± 0.20 (p=0.100)
PMA 100×	0.65 ± 0.60 (p=0.421)	0.61 ± 0.56 (p=0.351)	0.61 ± 0.53 (p=0.328)	0.40 ± 0.36 (p=0.100)
PP2 10×	1.13 ± 0.27 (p=0.482)	0.94 ± 0.11 (p=0.455)	1.03 ± 0.22 (p=0.819)	0.82 ± 0.30 (p=0.417)
PP2 100×	1.34 ± 0.57 (p=0.412)	1.02 ± 0.22 (p=0.875)	1.04 ± 0.06 (p=0.333)	0.99 ± 0.04 (p=0.725)
Ro31-8220 10×	0.70 ± 0.28 (p=0.199)	0.70 ± 0.23 (p=0.152)	0.92 ± 0.18 (p=0.504)	0.96 ± 0.18 (p=0.741)
Ro31-8220 100×	0.80 ± 0.31 (p=0.388)	0.72 ± 0.16 (p=0.095)	0.88 ± 0.09 (p=0.140)	0.75 ± 0.13 (p=0.082)
Rolipram 10×	0.82 ± 0.06 (p=0.031)	0.94 ± 0.16 (p=0.583)	1.07 ± 0.08 (p=0.237)	0.92 ± 0.03 (p=0.046)
Rolipram 100×	0.79 ± 0.19 (p=0.197)	0.84 ± 0.13 (p=0.174)	0.96 ± 0.10 (p=0.560)	0.82 ± 0.03 (p=0.007)
SNAP 10×	1.03 ± 0.20 (p=0.833)	0.94 ± 0.25 (p=0.732)	1.04 ± 0.15 (p=0.707)	1.00 ± 0.10 (p=0.986)
SNAP 100×	0.90 ± 0.36 (p=0.669)	0.79 ± 0.09 (p=0.050)	1.07 ± 0.16 (p=0.524)	1.07 ± 0.14 (p=0.458)
Sp-cAMPS 10×	1.20 ± 0.16 (p=0.168)	0.83 ± 0.11 (p=0.121)	1.18 ± 0.22 (p=0.304)	1.13 ± 0.28 (p=0.500)
Sp-cAMPS 100×	1.68 ± 0.37 (p=0.087)	1.10 ± 0.10 (p=0.239)	1.03 ± 0.28 (p=0.868)	0.78 ± 0.13 (p=0.106)
SQ22536 10×	1.22 ± 0.19 (p=0.185)	1.02 ± 0.17 (p=0.828)	0.92 ± 0.15 (p=0.443)	1.01 ± 0.09 (p=0.922)
SQ22536 100×	0.90 ± 0.44 (p=0.741)	1.07 ± 0.30 (p=0.730)	0.92 ± 0.25 (p=0.640)	1.14 ± 0.21 (p=0.362)
SU5416 10×	0.35 ± 0.05 (p=0.002)	0.76 ± 0.00 (p=0.000)	0.83 ± 0.06 (p=0.043)	0.94 ± 0.71 (p=0.902)
SU5416 100×	0.20 ± 0.06 (p=0.002)	0.62 ± 0.04 (p=0.004)	0.90 ± 0.31 (p=0.648)	0.99 ± 1.11 (p=0.990)
TAPI-1 10×	1.42 ± 0.05 (p=0.004)	0.76 ± 0.10 (p=0.047)	1.13 ± 0.05 (p=0.044)	1.14 ± 0.14 (p=0.230)
TAPI-1 100×	0.99 ± 0.45 (p=0.967)	0.62 ± 0.23 (p=0.101)	1.02 ± 0.28 (p=0.895)	0.93 ± 0.12 (p=0.426)
Taxol 10×	0.34 ± 0.13 (p=0.012)	0.56 ± 0.35 (p=0.164)	0.84 ± 0.41 (p=0.570)	0.60 ± 0.24 (p=0.102)
Taxol 100×	0.26 ± 0.13 (p=0.010)	0.32 ± 0.13 (p=0.012)	1.32 ± 0.56 (p=0.421)	0.87 ± 0.18 (p=0.357)
TGFβ RI inh 10×	0.94 ± 0.20 (p=0.633)	0.96 ± 0.16 (p=0.741)	1.04 ± 0.14 (p=0.655)	0.93 ± 0.09 (p=0.314)
TGFβ RI inh 100×	1.25 ± 0.12 (p=0.065)	1.23 ± 0.28 (p=0.296)	0.90 ± 0.11 (p=0.277)	0.90 ± 0.14 (p=0.314)
Vanadate 10×	1.44 ± 0.09 (p=0.015)	1.31 ± 0.25 (p=0.162)	1.15 ± 0.24 (p=0.394)	0.97 ± 0.09 (p=0.611)
Vanadate 100×	1.74 ± 1.49 (p=0.478)	0.84 ± 0.21 (p=0.316)	0.85 ± 0.11 (p=0.130)	0.80 ± 0.17 (p=0.180)
Y27632 10×	1.24 ± 0.23 (p=0.221)	1.05 ± 0.34 (p=0.825)	0.86 ± 0.08 (p=0.086)	0.81 ± 0.20 (p=0.243)
Y27632 100×	1.75 ± 0.17 (p=0.016)	1.38 ± 0.04 (p=0.004)	0.75 ± 0.03 (p=0.005)	0.86 ± 0.18 (p=0.313)
γ-sec inh X 10×	0.99 ± 0.34 (p=0.957)	0.94 ± 0.19 (p=0.636)	0.96 ± 0.06 (p=0.353)	0.89 ± 0.19 (p=0.429)
γ-sec inh X 100×	0.80 ± 0.45 (p=0.524)	0.75 ± 0.08 (p=0.033)	1.40 ± 0.31 (p=0.158)	1.13 ± 0.11 (p=0.164)
