# Supplementary material for: Quantitative assessment of angiogenesis and pericyte coverage in human cell-derived vascular sprouts
Source: Inflamm Regen. 2017 Jan 18;37:2. doi: 10.1186/s41232-016-0033-2 (PMC5725907; doi:10.1186/s41232-016-0033-2)
Supplement: Supplementary file 8 — Results of a pilot screen for EC/pericyte-derived vascular sprouts. Changes considered significant with a cut-off p value of p < 0.05 (two-sided one-sample t test compared to solvent control) are colored in red and green to indicate decrease and increase compared to controls, respectively. N = 3 assays with 8 wells per compound each. (PDF 67 kb) [file 41232_2016_33_MOESM8_ESM.pdf]

Results from a pilot screen for EC/pericyte sprouts

Condition	Number of sprouts (relative to control)	Sprout length (relative to control)	Total pericyte area (relative to control)	Pericyte area fraction (relative to control)
6-Bnz 10×	1.01 ± 0.19 (p=0.935)	0.95 ± 0.07 (p=0.350)	0.84 ± 0.55 (p=0.669)	0.98 ± 0.13 (p=0.834)
6-Bnz 100×	1.13 ± 0.53 (p=0.715)	0.79 ± 0.47 (p=0.516)	0.62 ± 0.10 (p=0.022)	0.82 ± 0.19 (p=0.240)
Blebbistatin 10×	1.05 ± 0.49 (p=0.869)	1.47 ± 0.21 (p=0.060)	1.83 ± 1.51 (p=0.440)	1.18 ± 0.18 (p=0.226)
Blebbistatin 100×	1.36 ± 0.65 (p=0.433)	0.97 ± 0.34 (p=0.894)	2.26 ± 0.91 (p=0.138)	2.15 ± 0.31 (p=0.023)
Calpeptin 10×	1.03 ± 0.02 (p=0.144)	0.94 ± 0.31 (p=0.770)	0.93 ± 0.40 (p=0.781)	1.20 ± 0.15 (p=0.145)
Calpeptin 100×	1.19 ± 0.37 (p=0.471)	1.11 ± 0.27 (p=0.561)	1.76 ± 0.30 (p=0.049)	1.35 ± 0.07 (p=0.012)
Calphostin C 10×	1.23 ± 0.23 (p=0.219)	1.07 ± 0.09 (p=0.303)	1.37 ± 0.32 (p=0.184)	1.12 ± 0.17 (p=0.352)
Calphostin C 100×	1.38 ± 0.65 (p=0.421)	1.10 ± 0.04 (p=0.042)	2.00 ± 0.87 (p=0.185)	1.26 ± 0.18 (p=0.127)
Cilostamide 10×	0.62 ± 0.27 (p=0.131)	0.79 ± 0.54 (p=0.560)	0.38 ± 0.44 (p=0.136)	0.74 ± 0.21 (p=0.162)
Cilostamide 100×	0.98 ± 0.36 (p=0.915)	0.93 ± 0.33 (p=0.744)	0.95 ± 0.10 (p=0.471)	1.15 ± 0.22 (p=0.344)
Cyclopamine 10×	1.09 ± 0.40 (p=0.730)	1.19 ± 0.23 (p=0.276)	1.04 ± 0.49 (p=0.911)	1.01 ± 0.14 (p=0.924)
Cyclopamine 100×	0.86 ± 0.46 (p=0.647)	1.44 ± 0.53 (p=0.288)	1.05 ± 1.20 (p=0.951)	1.28 ± 0.39 (p=0.350)
Cytochalasin D 10×	1.09 ± 0.32 (p=0.671)	0.45 ± 0.12 (p=0.014)	0.70 ± 0.09 (p=0.028)	1.06 ± 0.06 (p=0.224)
Cytochalasin D 100×	1.14 ± 1.02 (p=0.838)	0.42 ± 0.12 (p=0.014)	0.36 ± 0.06 (p=0.003)	0.96 ± 0.41 (p=0.893)
DAPT 10×	1.16 ± 0.38 (p=0.534)	1.22 ± 0.19 (p=0.187)	0.99 ± 0.43 (p=0.985)	0.91 ± 0.21 (p=0.533)
DAPT 100×	0.85 ± 0.62 (p=0.718)	0.69 ± 0.16 (p=0.082)	0.73 ± 0.50 (p=0.458)	1.07 ± 0.42 (p=0.801)
DDA 10×	1.06 ± 0.20 (p=0.637)	1.19 ± 0.06 (p=0.033)	1.61 ± 0.15 (p=0.020)	1.35 ± 0.44 (p=0.304)
DDA 100×	1.19 ± 0.36 (p=0.459)	1.26 ± 0.13 (p=0.076)	1.96 ± 1.45 (p=0.371)	1.49 ± 0.56 (p=0.274)
EHNA 10×	0.96 ± 0.04 (p=0.194)	0.93 ± 0.28 (p=0.707)	1.01 ± 0.37 (p=0.960)	1.09 ± 0.14 (p=0.386)
EHNA 100×	0.84 ± 0.27 (p=0.408)	0.71 ± 0.39 (p=0.324)	0.69 ± 0.55 (p=0.429)	0.98 ± 0.50 (p=0.950)
Eph inh 6 10×	1.33 ± 0.81 (p=0.552)	0.90 ± 0.18 (p=0.442)	1.38 ± 1.15 (p=0.628)	1.14 ± 0.29 (p=0.482)
Eph inh 6 100×	0.72 ± 0.57 (p=0.478)	0.85 ± 0.14 (p=0.212)	0.36 ± 0.28 (p=0.057)	0.68 ± 0.21 (p=0.123)
Eph inh 9 10×	0.80 ± 0.23 (p=0.266)	0.70 ± 0.22 (p=0.141)	0.55 ± 0.09 (p=0.012)	0.82 ± 0.27 (p=0.384)
Eph inh 9 100×	1.10 ± 0.16 (p=0.377)	0.81 ± 0.14 (p=0.134)	0.99 ± 0.73 (p=0.976)	1.04 ± 0.42 (p=0.882)
Forskolin 10×	1.18 ± 0.41 (p=0.523)	1.00 ± 0.24 (p=0.977)	1.39 ± 0.44 (p=0.264)	1.25 ± 0.32 (p=0.303)
Forskolin 100×	1.23 ± 0.39 (p=0.414)	1.02 ± 0.43 (p=0.952)	1.13 ± 0.45 (p=0.660)	1.19 ± 0.37 (p=0.466)
GM6001 10×	0.75 ± 0.55 (p=0.519)	0.61 ± 0.32 (p=0.172)	0.38 ± 0.49 (p=0.158)	0.93 ± 0.89 (p=0.899)
GM6001 100×	1.38 ± 0.22 (p=0.098)	0.65 ± 0.32 (p=0.193)	0.60 ± 0.49 (p=0.294)	1.09 ± 0.23 (p=0.559)
Gö-6976 10×	1.46 ± 0.90 (p=0.470)	0.72 ± 0.16 (p=0.091)	1.16 ± 0.41 (p=0.565)	1.28 ± 0.04 (p=0.006)
Gö-6976 100×	0.94 ± 0.44 (p=0.846)	0.80 ± 0.21 (p=0.235)	1.34 ± 0.82 (p=0.552)	1.62 ± 0.59 (p=0.207)
Gö-6983 10×	0.79 ± 0.25 (p=0.287)	0.67 ± 0.30 (p=0.202)	0.47 ± 0.30 (p=0.093)	0.84 ± 0.27 (p=0.413)
Gö-6983 100×	0.50 ± 0.59 (p=0.276)	0.61 ± 0.25 (p=0.119)	0.31 ± 0.37 (p=0.086)	1.40 ± 0.43 (p=0.242)
GSK3b inh IX 10×	0.99 ± 0.62 (p=0.985)	1.11 ± 0.23 (p=0.519)	0.82 ± 0.43 (p=0.552)	0.77 ± 0.35 (p=0.365)
GSK3b inh IX 100×	0.39 ± 0.04 (p=0.027)	1.47 ± 0.73 (p=0.532)	0.05 ± 0.07 (p=0.035)	0.34 ± 0.48 (p=0.301)
H-89 10×	0.72 ± 0.04 (p=0.008)	1.21 ± 0.33 (p=0.399)	0.73 ± 0.34 (p=0.295)	0.84 ± 0.35 (p=0.508)
H-89 100×	1.11 ± 0.15 (p=0.328)	0.96 ± 0.07 (p=0.442)	0.88 ± 0.57 (p=0.760)	0.84 ± 0.36 (p=0.510)
H1152 10×	1.05 ± 0.15 (p=0.614)	0.97 ± 0.05 (p=0.455)	0.96 ± 0.23 (p=0.767)	0.85 ± 0.11 (p=0.135)
H1152 100×	0.95 ± 0.50 (p=0.874)	1.07 ± 0.04 (p=0.077)	0.99 ± 0.72 (p=0.976)	0.83 ± 0.20 (p=0.278)
IBMX 10×	1.04 ± 0.37 (p=0.863)	1.08 ± 0.28 (p=0.680)	1.16 ± 0.99 (p=0.805)	1.09 ± 0.39 (p=0.732)
IBMX 100×	1.22 ± 0.80 (p=0.685)	0.91 ± 0.47 (p=0.782)	0.72 ± 0.28 (p=0.224)	1.40 ± 0.82 (p=0.491)
Jasplakinolide 10×	1.19 ± 0.75 (p=0.708)	0.91 ± 0.27 (p=0.614)	1.19 ± 0.95 (p=0.761)	0.94 ± 0.03 (p=0.082)
Jasplakinolide 100×	0.47 ± 0.64 (p=0.282)	0.22 ± 0.20 (p=0.020)	0.14 ± 0.20 (p=0.017)	0.82 ± 0.94 (p=0.766)

Results from a pilot screen for EC/pericyte sprouts

Condition	Number of sprouts (relative to control)	Sprout length (relative to control)	Total pericyte area (relative to control)	Pericyte area fraction (relative to control)
L-NOARG 10×	1.00 ± 0.52 (p=0.997)	1.01 ± 0.16 (p=0.890)	0.76 ± 0.19 (p=0.163)	0.75 ± 0.30 (p=0.290)
L-NOARG 100×	0.60 ± 0.26 (p=0.116)	0.87 ± 0.19 (p=0.351)	0.33 ± 0.20 (p=0.029)	0.95 ± 0.31 (p=0.794)
Latrunculin B 10×	0.97 ± 0.91 (p=0.966)	0.69 ± 0.06 (p=0.012)	0.59 ± 0.20 (p=0.068)	1.03 ± 0.19 (p=0.824)
Latrunculin B 100×	1.43 ± 1.95 (p=0.739)	0.37 ± 0.33 (p=0.080)	0.77 ± 1.25 (p=0.781)	0.73 ± 0.94 (p=0.669)
ML-7 10×	1.01 ± 0.34 (p=0.963)	0.96 ± 0.09 (p=0.482)	0.97 ± 0.07 (p=0.557)	1.06 ± 0.21 (p=0.648)
ML-7 100×	0.73 ± 0.96 (p=0.680)	0.76 ± 0.18 (p=0.144)	1.07 ± 1.53 (p=0.941)	1.57 ± 0.30 (p=0.080)
NFkB inh 10×	1.35 ± 1.68 (p=0.752)	0.36 ± 0.06 (p=0.003)	0.45 ± 0.55 (p=0.225)	1.34 ± 0.50 (p=0.367)
NFkB inh 100×	0.74 ± 0.64 (p=0.558)	0.56 ± 0.22 (p=0.072)	0.28 ± 0.07 (p=0.003)	1.37 ± 0.68 (p=0.446)
NKH477 10×	0.89 ± 0.35 (p=0.638)	0.70 ± 0.33 (p=0.249)	0.81 ± 0.58 (p=0.632)	0.99 ± 0.31 (p=0.961)
NKH477 100×	1.09 ± 0.52 (p=0.795)	0.51 ± 0.14 (p=0.027)	0.47 ± 0.31 (p=0.097)	0.95 ± 0.69 (p=0.919)
Nocodazole 10×	0.70 ± 1.00 (p=0.660)	0.19 ± 0.14 (p=0.009)	0.15 ± 0.23 (p=0.024)	0.81 ± 0.35 (p=0.446)
Nocodazole 100×	0.91 ± 1.31 (p=0.918)	0.20 ± 0.02 (p=0.000)	0.15 ± 0.14 (p=0.009)	1.08 ± 0.53 (p=0.819)
PDGFR inh IV 10×	0.63 ± 1.01 (p=0.595)	0.54 ± 0.54 (p=0.280)	0.65 ± 1.00 (p=0.605)	1.26 ± 0.96 (p=0.689)
PDGFR inh IV 100×	0.39 ± 0.62 (p=0.231)	0.95 ± 1.55 (p=0.964)	0.10 ± 0.13 (p=0.007)	0.87 ± 1.14 (p=0.865)
PMA 10×	1.13 ± 0.08 (p=0.121)	1.31 ± 0.59 (p=0.461)	2.30 ± 1.46 (p=0.264)	1.37 ± 0.14 (p=0.045)
PMA 100×	1.43 ± 0.70 (p=0.396)	1.26 ± 0.48 (p=0.445)	2.36 ± 1.79 (p=0.319)	1.25 ± 0.24 (p=0.213)
PP2 10×	1.32 ± 0.40 (p=0.302)	0.93 ± 0.16 (p=0.509)	1.01 ± 0.41 (p=0.960)	0.93 ± 0.13 (p=0.436)
PP2 100×	0.76 ± 0.06 (p=0.019)	0.86 ± 0.16 (p=0.271)	0.37 ± 0.07 (p=0.004)	0.80 ± 0.30 (p=0.369)
Ro-31-8220 10×	1.13 ± 0.43 (p=0.652)	0.78 ± 0.25 (p=0.265)	1.05 ± 0.72 (p=0.907)	1.12 ± 0.52 (p=0.723)
Ro-31-8220 100×	1.24 ± 1.02 (p=0.723)	0.45 ± 0.16 (p=0.029)	0.47 ± 0.57 (p=0.246)	0.60 ± 0.62 (p=0.377)
Rolipram 10×	1.07 ± 0.15 (p=0.520)	1.04 ± 0.27 (p=0.842)	1.56 ± 1.24 (p=0.518)	0.98 ± 0.45 (p=0.934)
Rolipram 100×	1.01 ± 0.33 (p=0.951)	1.27 ± 0.82 (p=0.620)	1.05 ± 0.63 (p=0.895)	1.06 ± 0.31 (p=0.784)
SNAP 10×	1.43 ± 0.60 (p=0.342)	1.09 ± 0.11 (p=0.313)	1.32 ± 0.60 (p=0.447)	1.06 ± 0.09 (p=0.372)
SNAP 100×	1.22 ± 0.41 (p=0.449)	1.08 ± 0.25 (p=0.644)	1.34 ± 0.16 (p=0.066)	1.01 ± 0.20 (p=0.933)
Sp-cAMPS 10×	0.81 ± 0.19 (p=0.223)	1.04 ± 0.26 (p=0.816)	0.55 ± 0.54 (p=0.285)	0.58 ± 0.44 (p=0.240)
Sp-cAMPS 100×	1.14 ± 0.21 (p=0.361)	1.37 ± 0.30 (p=0.166)	1.00 ± 0.89 (p=0.997)	0.61 ± 0.55 (p=0.345)
SQ22536 10×	1.07 ± 0.15 (p=0.526)	0.99 ± 0.16 (p=0.918)	1.57 ± 0.18 (p=0.033)	1.20 ± 0.15 (p=0.155)
SQ22536 100×	1.16 ± 0.06 (p=0.042)	1.06 ± 0.26 (p=0.717)	1.11 ± 0.37 (p=0.655)	1.12 ± 0.06 (p=0.075)
SU5416 10×	0.87 ± 0.42 (p=0.645)	0.80 ± 0.13 (p=0.111)	1.27 ± 0.77 (p=0.608)	1.36 ± 0.69 (p=0.462)
SU5416 100×	0.52 ± 0.33 (p=0.128)	0.63 ± 0.24 (p=0.122)	0.22 ± 0.12 (p=0.007)	0.94 ± 0.14 (p=0.543)
TAPI-1 10×	1.66 ± 0.57 (p=0.183)	0.94 ± 0.25 (p=0.722)	1.57 ± 0.35 (p=0.108)	1.19 ± 0.12 (p=0.122)
TAPI-1 100×	1.46 ± 0.28 (p=0.102)	0.94 ± 0.12 (p=0.485)	1.61 ± 0.72 (p=0.278)	1.13 ± 0.55 (p=0.725)
Taxol 10×	1.17 ± 0.91 (p=0.774)	0.49 ± 0.08 (p=0.009)	0.42 ± 0.14 (p=0.018)	1.53 ± 0.68 (p=0.307)
Taxol 100×	0.51 ± 0.44 (p=0.193)	0.46 ± 0.21 (p=0.047)	0.17 ± 0.12 (p=0.007)	1.09 ± 0.47 (p=0.765)
TGFβ RI inh 10×	1.26 ± 0.29 (p=0.261)	1.08 ± 0.23 (p=0.603)	1.56 ± 0.32 (p=0.097)	1.27 ± 0.12 (p=0.062)
TGFβ RI inh 100×	1.76 ± 0.18 (p=0.019)	0.81 ± 0.18 (p=0.216)	1.51 ± 0.74 (p=0.357)	1.08 ± 0.47 (p=0.803)
Vanadate 10×	1.17 ± 0.33 (p=0.470)	1.01 ± 0.14 (p=0.950)	1.17 ± 0.44 (p=0.569)	0.99 ± 0.28 (p=0.949)
Vanadate 100×	1.07 ± 1.63 (p=0.947)	0.26 ± 0.34 (p=0.063)	0.79 ± 1.35 (p=0.813)	0.76 ± 1.18 (p=0.761)
Y27632 10×	1.35 ± 0.32 (p=0.205)	0.85 ± 0.16 (p=0.256)	0.77 ± 0.21 (p=0.190)	0.89 ± 0.22 (p=0.463)
Y27632 100×	1.13 ± 0.14 (p=0.246)	0.88 ± 0.16 (p=0.312)	1.04 ± 0.30 (p=0.845)	0.92 ± 0.18 (p=0.533)
γ-sec inh X 10×	0.73 ± 0.22 (p=0.165)	1.10 ± 0.36 (p=0.678)	0.53 ± 0.31 (p=0.121)	0.59 ± 0.40 (p=0.221)
γ-sec inh X 100×	1.32 ± 0.13 (p=0.048)	0.85 ± 0.34 (p=0.516)	1.37 ± 0.75 (p=0.481)	1.05 ± 0.39 (p=0.831)
