# Supplementary material for: Quantitative assessment of angiogenesis and pericyte coverage in human cell-derived vascular sprouts
Source: Inflamm Regen. 2017 Jan 18;37:2. doi: 10.1186/s41232-016-0033-2 (PMC5725907; doi:10.1186/s41232-016-0033-2)
Supplement: Supplementary file 9 — Comparison of in vitro and ex vivo assays. Comparison of in vitro and ex vivo assays to study sprouting angiogenesis, EC migration, and pericyte coverage. The method presented here is highlighted in green. Symbols used are the following: +, possible/present; −, not possible/not present; n.s., not shown. (DOCX 148 kb) [file 41232_2016_33_MOESM9_ESM.docx]

| **Assay** | **Time frame** | **Biological question** | **Vascular network** | **Sprouting** | **Human cells** | **Blood flow** | **Presence of pericytes** | **Pericyte coverage quant.** | **Sprout width quant.** | **Use of lymphatic cells** | **Cancer cells as feeders** | **Multi-well scale possible** |
| --- | --- | --- | --- | --- | --- | --- | --- | --- | --- | --- | --- | --- |
| **In vitro assays** |  |  |  |  |  |  |  |  |  |  |  |  |
| In vitro scratch assay^90^ | 10 – 24 h | Cell migration | — | — | + | — | — | — | — | n.s. | — | + |
| Chemoinvasion assay^91^ | 9 h | EC invasion | — | — | + | — | — | — | — | n.s. | — | + |
| Invasion and sprouting in collagen^92^ | 24 h | Sprout formation and initiation | + | + | + | — | — | — | n.s. | n.s. | — | + |
| RAIN-droplet assay^93^ | 3 – 6 d | 3D focal sprouting, cell migration and invasion | + | + | + | — | — | — | n.s. | — | — | + |
| Tube formation on gelled basement membrane extract^94^ | 4 – 18 h | EC adhesion, migration, protease activity, tubule formation | + | — | + | — | — | — | n.s. | + | — | + |
| Spheroid-based sprouting assay^95^ | 3 d | Sprouting of lumenized capillary-like structures | + | + | + | — | n.s. | n.s. | n.s. | n.s. | n.s. | + |
| Microvascular networks in vitro^96^ | 1 – 14 d | Tumor angiogenesis | + | — | + | + | + | n.s. | — | — | + | — |
| 3D culture inside a microfluidic device^97^ | 12 – 24 h | EC/pericyte interaction in a perfused 3D enviroment | — | + | + | + | + | n.s. | — | — | — | — |
| Embryoid bodies^98-100^ | 5 – 16 d | Vasculogenesis, sprouting angiogenesis | + | + | — | — | + | n.s. | n.s. | n.s. | — | + |
| Fibrin bead sprout­ing assay^101-104^ | 5 – 20 d | Vascular sprouting angiogenesis, pericyte coverage | — | + | + | — | + | + | + | + | + | + |
| **Ex vivo assays** |  |  |  |  |  |  |  |  |  |  |  |  |
| Aortic ring assay^105,106^ | 6 – 14 d | Vascular sprouting angiogenesis | — | + | — | — | + | + | — | — | — | + |
| Choroid sprouting assay^107^ | 4 – 6 d | Vascular sprouting angiogenesis | — | + | — | — | + | n.s. | — | — | — | — |
| Retinal explant cultures^108^ | 10 h | Vascular sprouting angiogenesis | + | + | — | — | + | n.s. | — | — | — | — |
| Ex vivo-cultured mouse embryos^109^ | 3 – 5 d | Vasculogenesis, sprouting angiogenesis, lymphatic development and sprouting | + | + | — | + | + | n.s. | n.s. | n.s. | — | — |

References

90. Liang, C.C., Park, A.Y. & Guan, J.L. In vitro scratch assay: a convenient and inexpensive method for analysis of cell migration in vitro. *Nature protocols* **2**, 329-333 (2007).

91. Albini, A. & Benelli, R. The chemoinvasion assay: a method to assess tumor and endothelial cell invasion and its modulation. *Nature protocols* **2**, 504-511 (2007).

92. Bayless, K.J., Kwak, H.I. & Su, S.C. Investigating endothelial invasion and sprouting behavior in three-dimensional collagen matrices. *Nature protocols* **4**, 1888-1898 (2009).

93. Zeitlin, B.D., Dong, Z. & Nor, J.E. RAIN-Droplet: a novel 3D in vitro angiogenesis model. *Laboratory investigation; a journal of technical methods and pathology* **92**, 988-998 (2012).

94. Arnaoutova, I. & Kleinman, H.K. In vitro angiogenesis: endothelial cell tube formation on gelled basement membrane extract. *Nature protocols* **5**, 628-635 (2010).

95. Korff, T. & Augustin, H.G. Tensional forces in fibrillar extracellular matrices control directional capillary sprouting. *Journal of cell science* **112 ( Pt 19)**, 3249-3258 (1999).

96. Morgan, J.P.*, et al.* Formation of microvascular networks in vitro. *Nature protocols* **8**, 1820-1836 (2013).

97. van der Meer, A.D., Orlova, V.V., ten Dijke, P., van den Berg, A. & Mummery, C.L. Three-dimensional co-cultures of human endothelial cells and embryonic stem cell-derived pericytes inside a microfluidic device. *Lab on a chip* **13**, 3562-3568 (2013).

98. Magnusson, P.*, et al.* Deregulation of Flk-1/vascular endothelial growth factor receptor-2 in fibroblast growth factor receptor-1-deficient vascular stem cell development. *Journal of cell science* **117**, 1513-1523 (2004).

99. Jakobsson, L.*, et al.* Heparan sulfate in trans potentiates VEGFR-mediated angiogenesis. *Developmental cell* **10**, 625-634 (2006).

100. Jakobsson, L.*, et al.* Endothelial cells dynamically compete for the tip cell position during angiogenic sprouting. *Nature cell biology* **12**, 943-953 (2010).

101. Nakatsu, M.N.*, et al.* Angiogenic sprouting and capillary lumen formation modeled by human umbilical vein endothelial cells (HUVEC) in fibrin gels: the role of fibroblasts and Angiopoietin-1. *Microvascular research* **66**, 102-112 (2003).

102. Nakatsu, M.N., Davis, J. & Hughes, C.C. Optimized fibrin gel bead assay for the study of angiogenesis. *Journal of visualized experiments : JoVE*, 186 (2007).

103. Nakatsu, M.N. & Hughes, C.C. An optimized three-dimensional in vitro model for the analysis of angiogenesis. *Methods in enzymology* **443**, 65-82 (2008).

104. Brudno, Y., Ennett-Shepard, A.B., Chen, R.R., Aizenberg, M. & Mooney, D.J. Enhancing microvascular formation and vessel maturation through temporal control over multiple pro-angiogenic and pro-maturation factors. *Biomaterials* **34**, 9201-9209 (2013).

105. Aplin, A.C., Fogel, E., Zorzi, P. & Nicosia, R.F. The aortic ring model of angiogenesis. *Methods in enzymology* **443**, 119-136 (2008).

106. Baker, M.*, et al.* Use of the mouse aortic ring assay to study angiogenesis. *Nature protocols* **7**, 89-104 (2012).

107. Shao, Z.*, et al.* Choroid sprouting assay: an ex vivo model of microvascular angiogenesis. *PloS one* **8**, e69552 (2013).

108. Sawamiphak, S., Ritter, M. & Acker-Palmer, A. Preparation of retinal explant cultures to study ex vivo tip endothelial cell responses. *Nature protocols* **5**, 1659-1665 (2010).

109. Zeeb, M.*, et al.* Pharmacological manipulation of blood and lymphatic vascularization in ex vivo-cultured mouse embryos. *Nature protocols* **7**, 1970-1982 (2012).
