# Supplementary figures and images for: Quantitative assessment of angiogenesis and pericyte coverage in human cell-derived vascular sprouts
Source: Inflamm Regen. 2017 Jan 18;37:2. doi: 10.1186/s41232-016-0033-2 (PMC5725907; doi:10.1186/s41232-016-0033-2)

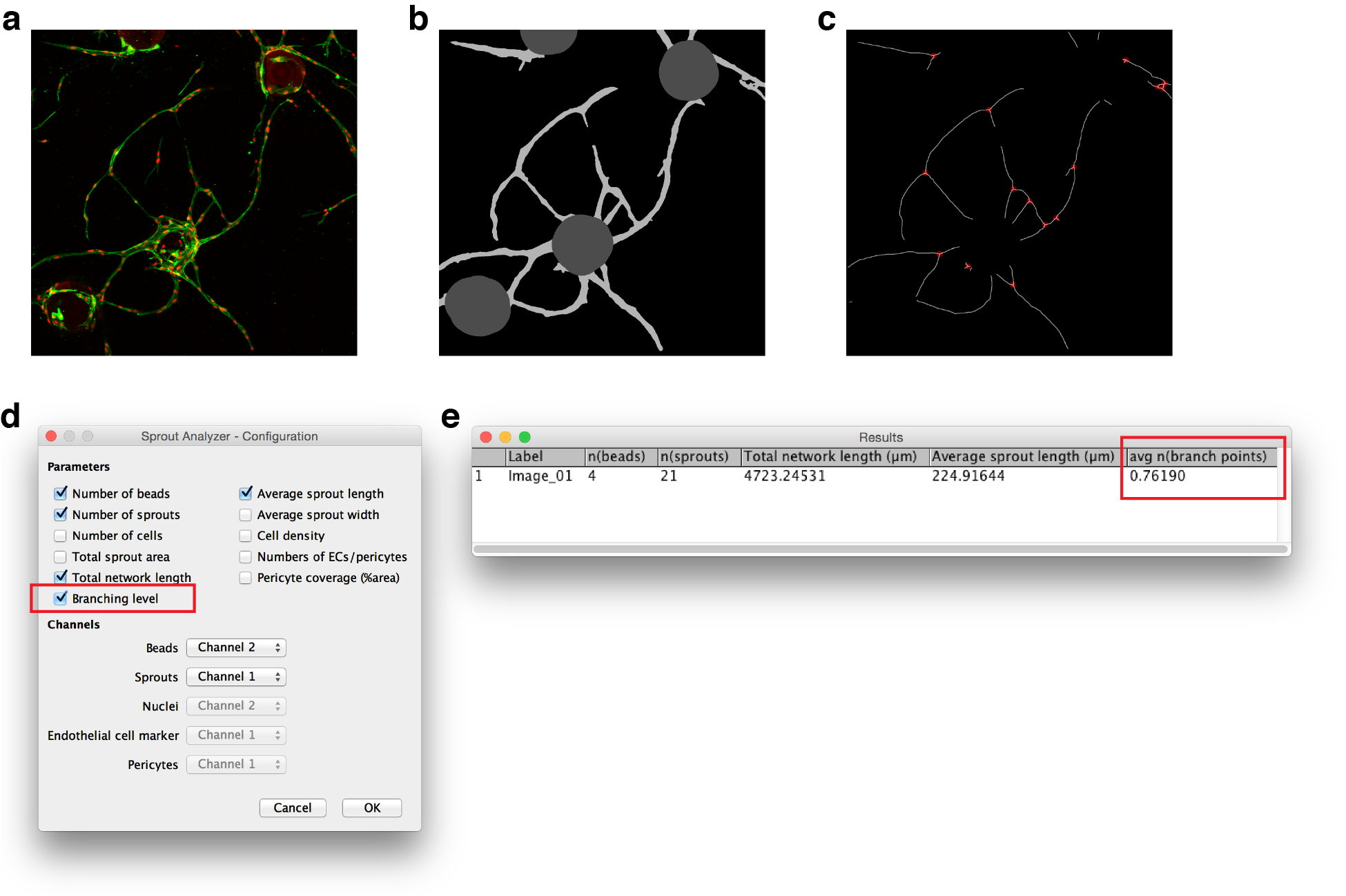

Supplement: Supplementary file 10 — Quantification of branching level. (a) Confocal micrograph (maximum intensity projection) of a sprouting vascular plexus growing from four microcarrier beads, stained for F-actin (green) and nuclei (red). (b) Result image showing bead (dark grey) and sprout (light grey) segmentation. (c) Result image showing the sprout skeletons; branch points have been highlighted in red. (d) The option to measure branching level (red box) is available in the configuration dialog. (e) Result table reporting the average number of branch points per sprout (avg n(branch points), red box). (PNG 375 kb) [file 41232_2016_33_MOESM10_ESM.png]
